# MetalCenter‐Dependent Selectivity Divergence in MN_4_ Single‐Atom Catalysts for Aerobic HMF Oxidation

**DOI:** 10.1002/advs.202524182

**Published:** 2026-04-21

**Authors:** Haoyu Wang, Zhilong Ye, Weihao Wang, Zhi Hu, Qing Tian, Jie Gao, Lianbing Zhang

**Affiliations:** ^1^ School of Life Science and Technology Northwestern Polytechnical University Xi'an P. R. China; ^2^ Zhejiang Key Laboratory of Low‐carbon Control Technology for Industrial Pollution College of Environment Zhejiang University of Technology Hangzhou P. R. China; ^3^ Nanozyme Laboratory in Zhongyuan Henan Academy of Innovations in Medical Science Zhengzhou P. R. China; ^4^ Nanozyme Laboratory in Zhongyuan College of Chemistry and Molecular Sciences Henan University Zhengzhou Henan P. R. China

**Keywords:** 5‐Hydroxymethylfurfural, aerobic oxidation, electronic regulation, selectivity, single‐atom catalysts

## Abstract

Selective oxidation of carbohydrate‐derived 5‐hydroxymethylfurfural (HMF) to high‐value chemicals is a critical pathway for sustainable development. However, the primary challenge is controlling selectivity, as the narrow energy gaps between intermediates make discriminating between specific functional groups difficult. Herein, two single‐atom catalysts (SACs) with M‐N_4_ configurations were synthesized by varying the metal active center. The FeN_4_ catalyst achieved 99.9% HMF conversion with 93.9% selectivity toward 2‐formyl‐5‐furancarboxylic acid (FFCA) at 333.15 K. Conversely, the CoN_4_ catalyst yielded 2,5‐furandicarboxylic acid (FDCA) as the predominant product with comparable HMF conversion. Despite divergent product selectivities, the two catalysts displayed nearly identical TOFs for HMF consumption (∼18.2 h^−1^). DFT calculations revealed that the metal center governs the adsorption/activation of the FFCA intermediate. Specifically, lower FFCA adsorption energy on FeN_4_ inhibits dehydrogenation at the *α*‐C position, while stronger adsorption on CoN_4_ facilitates its subsequent oxidation to FDCA. Moreover, the catalysts showed distinct O_2_ activation mechanisms. FeN_4_ preferentially generates superoxide anions (O_2_^•−^) for HMF oxidation, while CoN_4_ utilizes hydroxyl radicals (•OH) for FFCA oxidation to FDCA. This study demonstrates an effective strategy for controlled HMF oxidation, providing a theoretical basis for tailoring reaction selectivity through a mechanistic divergence driven by the intrinsic electronic structure of the metal center.

## Graphical abstract

Two single‐atom catalysts with M‐N_4_ configurations (FeN_4_ and CoN_4_) were developed for selective HMF oxidation. FeN_4_ achieves 93.9% selectivity toward FFCA via superoxide anions (O_2_^•−^), while CoN_4_ preferentially produces FDCA through hydroxyl radicals (•OH). DFT calculations reveal that distinct adsorption energies and charge redistribution at the metal centers govern the divergent selectivity, offering a strategy for tunable biomass upgrading.

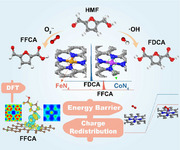

## 1 Introduction

The transition from fossil resources to sustainable biomass to produce high‐value chemicals is a central objective of modern chemistry. 5‐Hydroxymethylfurfural (HMF) is a versatile platform molecule, designated by the US Department of Energy as one of the top ten bio‐based chemicals for future industrial applications [1]. However, the industrial valorization of HMF is often hindered by the inherent instability of its furanic core and the competitive oxidation of its primary functional groups, which frequently leads to complex product distributions, over‐oxidation, and poor selectivity toward targeted value‐added products [2, 3, 4]. These functional groups exhibit high chemical reactivity and undergo various transformations, including oxidation, hydrogenation, and condensation, to yield diverse high‐value chemicals [5]. Thus, the precise control of HMF oxidation to yield a specific product over a complex mixture remains a pivotal challenge. Solving this requires well‐defined catalytic architectures and a fundamental understanding of how the active center's electronic state orchestrates intermediate adsorption and oxygen activation, ultimately driving the selectivity toward either partial or deep oxidation.

In recent decades, various strategies, including photocatalysis, electrocatalysis, biocatalysis, and aerobic oxidation, have been developed for the selective conversion of HMF. Aerobic oxidation has emerged as the preferred pathway for selective HMF oxidation, particularly for industrial FDCA production, owing to its cost‐effectiveness, environmental compatibility, and suitability for continuous processing. Although noble metals (Pt, Au, and Pd) are effective catalysts for selective FDCA production, their high cost and variable selectivity restrict their practical applications [6, 7, 8]. Non‐noble metal catalysts are cost‐effective alternatives with satisfactory HMF conversion activities. However, product selectivity under mild conditions remains inadequate [9]. Single‐atom catalysts (SACs), which feature isolated metal atoms stabilized on support, have attracted considerable attention for biomass‐derived platform chemical conversion due to their maximal atomic utilization and reliable selectivity [10, 11, 12, 13]. For instance, lignin‐based cobalt SACs specifically catalyze the conversion of HMF to FDCA under mild conditions, demonstrating the unique properties of SACs in selective HMF oxidation [14]. Despite these advances, current SAC designs predominantly favor the oxidation of aldehyde groups, and a generalizable strategy for switching selectivity among different partially or fully oxidized products of HMF remains elusive.

The distinctive M‐N_4_ sites in nitrogen‐doped carbon‐supported SACs, characterized by their structural precision and well‐defined coordination environment, feature tetrahedral nitrogen coordination and tunable electronic structures that operate according to the Sabatier principle for oxygen activation [15, 16, 17]. These characteristics are suitable for the precise modulation of the selective oxidation of HMF. Their adjustable coordination configurations and atomic structures provide an effective strategy for tailoring catalytic activity and selectivity. Research has established that the reaction selectivity and product distribution depend on the relative activation energies of different pathways, which correlate directly with the electronic structure of the catalytically active sites [18]. The M‐N_4_ motifs embedded in carbon matrices constitute prototypical and well‐defined structures. The electronic structure of the metal centers within the M‐N_4_ sites serves as a critical catalytic performance descriptor that governs the reactant adsorption, oxygen activation, and elementary step energy barriers [15, 16, 19]. Based on these findings, we hypothesized that precise control of HMF oxidation selectivity can be achieved by modulating the electronic structure of isolated metal centers in M‐N_4_ configurations, utilizing differences in *d*‐orbital occupancy to regulate the intermediate binding affinities and reaction barriers.

Herein, a metal‐site substitution strategy was employed to create M‐N_4_ (M = Fe, Co) configurations with similar coordination environments but distinct electronic structures (e.g., Co^2+^ with partially filled *e_g_* orbitals vs. Fe^2+^ with *t_2g_*‐dominated states), ultimately governing the HMF selective oxidation pathway [20]. Experimentally, the FeN_4_ catalyst selectively oxidized HMF to FFCA with an outstanding yield of 93.9% at an HMF conversion exceeding 99.9%, whereas the CoN_4_ catalyst promoted the near‐complete oxidation of HMF to FDCA, achieving an exceptional yield of 99.0% with a conversion rate of over 99.9%. Spin‐polarized DFT calculations and projected Crystal Orbital Hamilton Population (pCOHP) analyses revealed that the FFCA adsorption energy is governed by M‐O covalency. The FeN_4_ site exhibits stronger Fe‐O covalency (higher ‐ICOHP), resulting in tighter FFCA binding and favoring intermediate desorption before deep oxidation. In contrast, the CoN_4_ site displays weaker Co‐O covalency (lower ‐ICOHP), enabling moderate FFCA adsorption that sustains the catalytic cycle toward FDCA. EPR spectroscopy and charge‐transfer analyses further confirm that distinct oxygen activation pathways at the M‐N_4_ sites critically govern the selective oxidation of HMF. This study is expected to provide new insights into the structure–activity relationship of SACs for complex selective oxidation reactions.

## 2 Results and Discussion

### 2.1 Synthesis and Characterization of SACs

**FIGURE 1:**
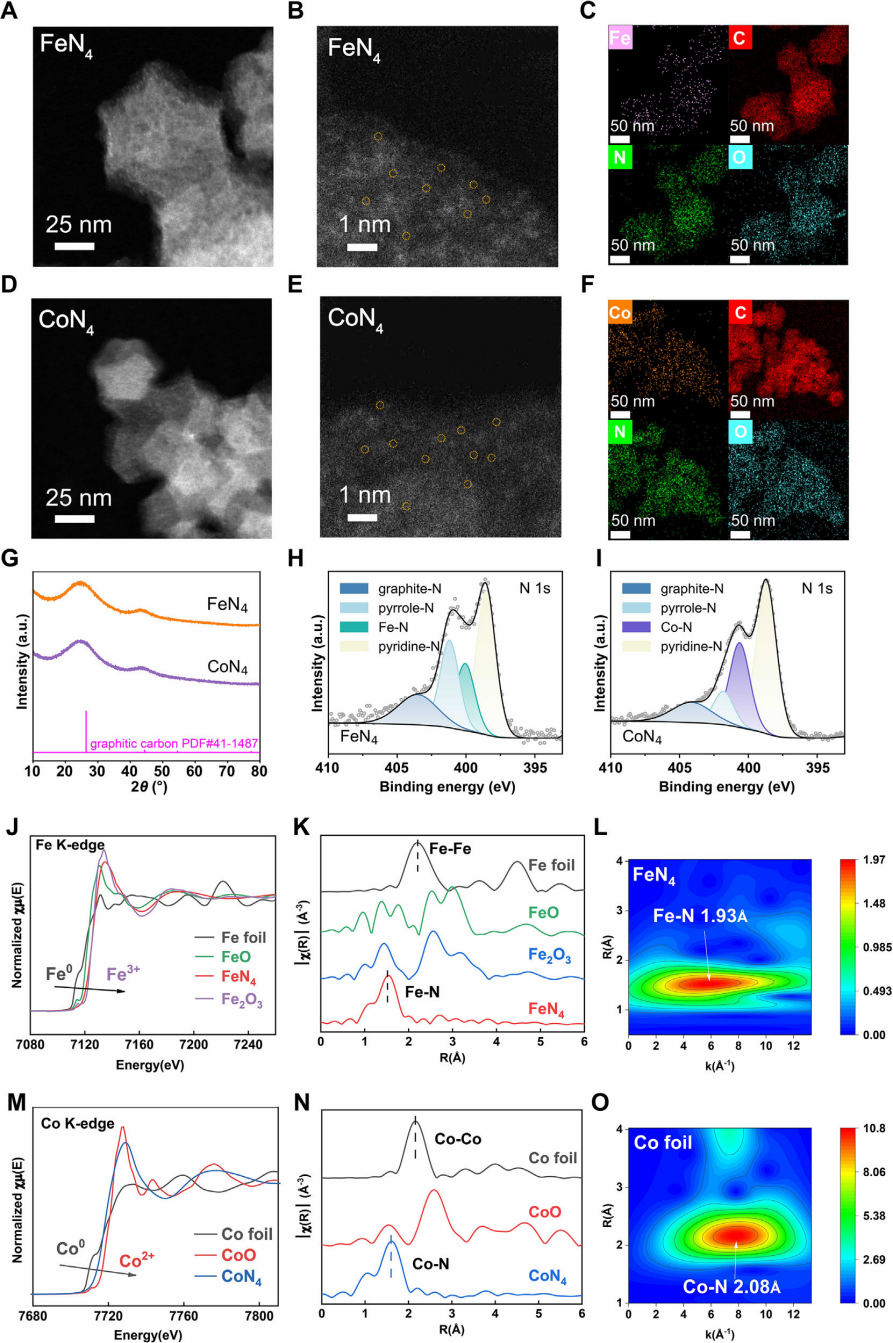
Structural and compositional characterizations of FeN_4_ and CoN_4_ single‐atom catalysts (SACs). (A,D) STEM and (B,E) atomic‐resolution HAADF‐STEM images of FeN_4_ and CoN_4_, respectively. (C,F) Corresponding EDS elemental mapping. (G) XRD patterns of as‐synthesized catalysts. (H,I) High‐resolution N 1s XPS spectra of the samples. (J,M) XANES spectra. (K,N) Fourier‐transformed EXAFS spectra. (L,O) Wavelet transforms for the catalysts and their corresponding metal foils.

Atomically dispersed iron and cobalt centers supported on nitrogen‐doped carbon were successfully synthesized through the pyrolysis of the corresponding metal‐containing zeolitic imidazolate framework (ZIF) precursors. TEM and HAADF‐STEM analyses (Figure 1A,D) revealed that the as‐prepared FeN_4_ and CoN_4_ catalysts completely retained the typical rhombic dodecahedral morphology of their ZIF precursors (Figure S1) [21]. Their carbon matrices showed only uniformly dispersed bright spots without any iron‐ or cobalt‐containing nanoparticles or clusters (Figure 1B,E), findings that were further corroborated by the XRD results (Figure 1G). Two broad diffraction features at approximately 25° and 44° were observed, which are characteristic of amorphous or graphitic carbon (PDF#41‐1487), indicating a relatively high degree of graphitization [22]. Energy‐dispersive X‐ray spectroscopy (EDS) analysis revealed that carbon, nitrogen, oxygen, and metal Fe/Co were homogeneously dispersed on the carbon matrix (Figure 1C,F). ICP‐OES quantification showed metal loadings of 0.99 wt. % for FeN_4_ and 2.03 wt. % for CoN_4_ (Table S1). As shown in Table S2, the BET surface areas of FeN_4_ and CoN_4_ were 502 and 573 m^2^ g^−1^, respectively. The similar BET surface areas suggest a negligible effect of the metal center identity on the specific surface area of M‐N‐C materials [23, 24]. Moreover, both FeN_4_ and CoN_4_ exhibit type IV adsorption–desorption isotherms with steep uptake at high relative pressure and pronounced hysteresis (Figure S2A,B). While CoN_4_ is predominantly microporous (pore size < 2 nm), FeN_4_ retains microporous characteristics while developing significant mesoporosity (Figure S2C,D). These textural differences originate from subtle variations in the carbonization and templating processes within the respective metal‐ZIF systems.

X‐ray photoelectron spectroscopy (XPS) was used to confirm the interaction between the metal and carbon matrices. The N 1s XPS spectra (Figure 1H,I) were deconvoluted into four peaks, representing pyridinic N, pyrrolic N, graphitic N, and a distinct M‐N_x_ peak. The presence of this M‐N_x_ component provided direct spectroscopic evidence of metal‐nitrogen coordination bond formation, confirming the successful anchoring of single metal atoms within the N‐doped carbon matrix. The slight differences in the binding energies of similar N‐species between the two samples are attributed to the distinct electronic environments created by the different metal centers: the Fe─N and Co─N coordination bonds exhibit different covalency and charge transfer characteristics, resulting in measurable shifts in the electron density of neighboring N atoms [25]. As seen from the X‐ray absorption near‐edge structure (XANES) spectra, the absorption energy of FeN_4_ was between that of the FeO and Fe_2_O_3_ reference materials (Figure 1J), demonstrating that the Fe oxidation state was in the range of Fe^2+^ and Fe^3+^ [26]. CoN_4_ exhibits a nearly identical first absorption edge position (E_0_) in its XANES spectrum compared to that of CoO (Figure 1M), indicating a dominant +2 valence state, though a minor contribution from Co^3+^ cannot be entirely excluded in the absence of a Co_2_O_3_ reference standard. The extended X‐ray absorption fine structure (EXAFS) spectrum confirmed the exclusive presence of Fe─N and Co─N bonds with no detectable metal‐metal contributions (Figure 1K,N), ruling out the formation of nanoparticles or clusters. The wavelet transform EXAFS (Figure 1L,O) displays single maxima at 1.93 and 2.08 Å corresponding to the Fe─N and Co─N scattering paths, respectively. Quantitative EXAFS fitting (Figures S3–S6 and Table S3) further determined coordination numbers of approximately 4 for both metal‐nitrogen bonds, unequivocally demonstrating the formation of M‐N_4_ configurations in these materials.

### 2.2 Selective Oxidation of HMF Catalyzed by SACs

**FIGURE 2:**
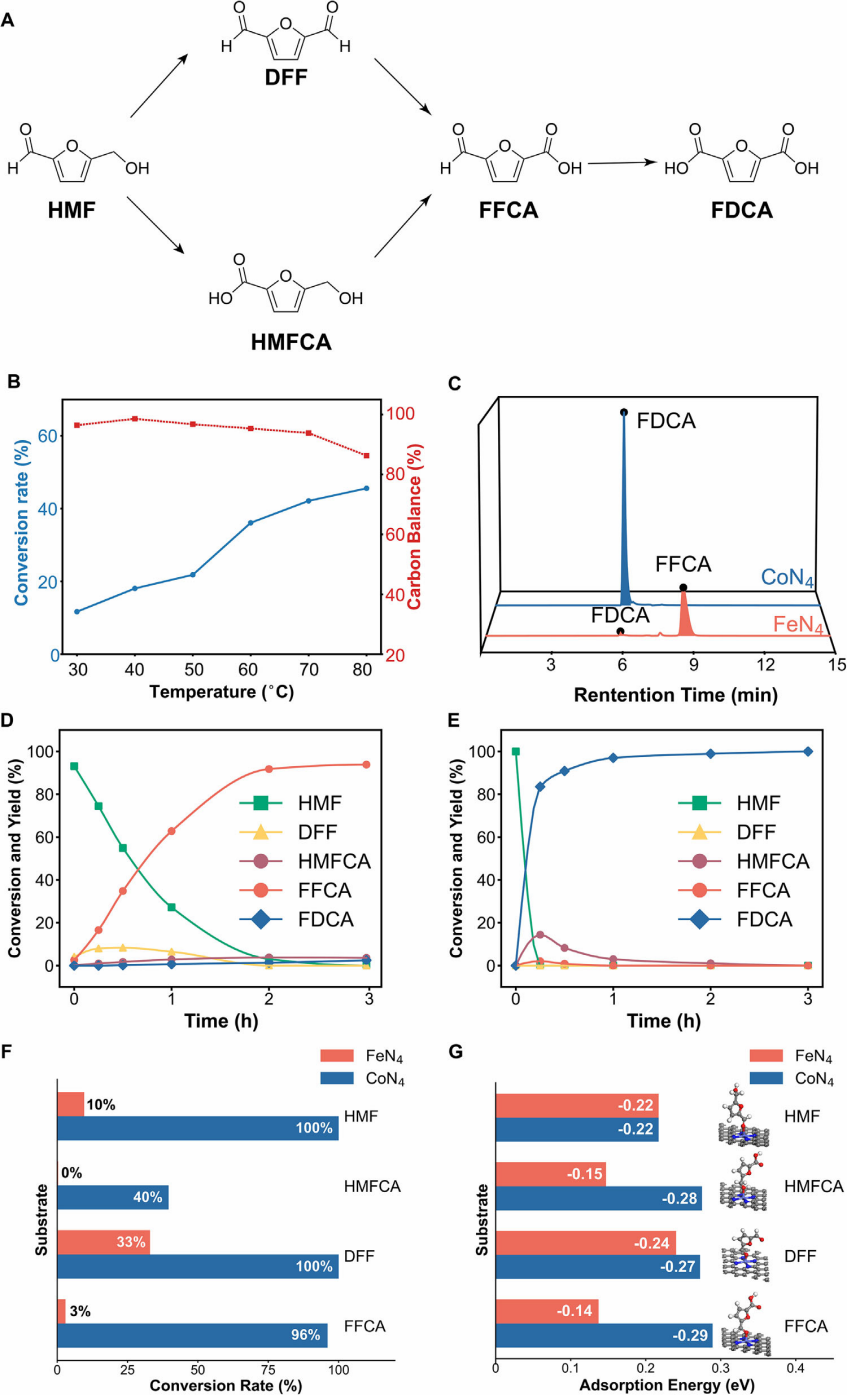
Catalytic performance and selectivity for HMF oxidation over FeN_4_ and CoN_4_ catalysts. (A) Reaction pathways for the oxidation of HMF to form FDCA. (B) Effect of reaction temperature on the HMF conversion rate (blue line) and carbon balance (red line) at an initial pH of 12.5 in 30 min. (C) HPLC profiles of the final products from the reactions over FeN_4_ and CoN_4_. Time course of HMF conversion and product distribution over (D) FeN_4_ and (E) CoN_4_. (F) Catalytic conversion rates of HMF, DFF, HMFCA, and FFCA substrates. (G) DFT‐calculated adsorption energies of HMF and its key intermediates on the different catalysts. Reaction conditions: 5 mmol of NaOH, 0.5 mmol of HMF, 40 mg of catalyst, and 100 mL of H_2_O were added to the reactor with a continuous O_2_ flow of 50 mL/min, stirring rate = 1200 rpm).

The aerobic oxidation of HMF proceeded through a cascade reaction via two canonical pathways that ultimately converged at the FFCA (Figure 2A) [27, 28]. The first pathway involves the selective oxidation of the primary alcohol group in HMF to yield DFF, which subsequently undergoes oxidation to FFCA. The second pathway begins with the oxidation of the aldehyde group to form HMFCA, followed by alcohol oxidation to yield FFCA. The final and most challenging step involves the oxidation of the remaining aldehyde group in FFCA to produce the fully oxidized target molecule, FDCA. First, standard curves (Figure S7) for all analytes were established using High‐Performance Liquid Chromatography (HPLC) to ensure quantification accuracy. Through systematic pH optimization, standard conditions (aqueous solution at pH 12.5) were obtained to maximize the HMF conversion while maintaining a high carbon balance (Figures S8 and S9). The slight decline in carbon balance observed at higher temperatures (e.g., 80°C) is likely due to the inherent instability of the HMF molecule in highly alkaline media, leading to minor base‐catalyzed degradation and the formation of soluble or insoluble humins. Considering factors such as reaction time and total carbon balance, we selected 60°C as the optimal temperature, even though the catalyst also exhibited considerable activity at 30°C (Figure 2B).

As illustrated in the condition optimization, a decline in the overall carbon balance was observed under highly alkaline conditions or at elevated substrate concentrations (Figures S10 and S11). Consistent with previous reports [14], this carbon deficit is primarily attributed to the base‐catalyzed degradation of unreacted HMF, which undergoes competitive aldol condensation and furan ring‐opening reactions to form soluble byproducts and oligomeric precursors (humins), rather than overoxidation to CO_2_. Under identical reaction conditions, the FeN_4_ and CoN_4_ catalysts exhibited striking divergence in product selectivity (Figure 2C). FeN_4_ demonstrated high selectivity for the partial oxidation of HMF, yielding FFCA (93.9% conversion) almost exclusively with only trace amounts of FDCA (2.5% conversion). In contrast, CoN_4_ displayed exceptional deep oxidation activity, converting HMF almost quantitatively to FDCA (99.0% conversion). The observed difference in product distribution is clear proof that the single metal atom identity in the SACs tunes the selective oxidation pathway of HMF. Arrhenius analysis of the temperature‐dependent rates yielded apparent activation energies of 28.45 kJ/mol for FeN_4_ and 8.22 kJ/mol for CoN_4_ for HMF (Figure S12). Notably, despite their markedly different product selectivities, the two catalysts exhibited comparable turnover frequencies (TOFs) for HMF consumption, with FeN_4_ and CoN_4_ affording TOF values of 18.12 and 18.42 h^−1^, respectively (Table S4). This similarity in intrinsic HMF conversion rates indicates that the divergence in selectivity does not originate from a gross difference in overall catalytic activity, but rather from the distinct ability of each metal center to activate specific functional groups and drive subsequent oxidation steps beyond the initial HMF transformation. While the lower reaction barrier for CoN_4_ generally corresponds to a more favorable kinetic environment, the exceptionally low *E_a_* value (< 10 kJ/mol) also suggests that the overall reaction rate on this highly active single‐atom catalyst may be partially influenced by diffusion‐limited processes, even though mass transfer resistance was minimized through optimized stirring and small catalyst particle size. A relatively low reaction barrier corresponds to a more favorable kinetic environment for the substrate conversion. To decipher how this kinetic advantage governs product selectivity, time‐resolved concentration profiles were further analyzed, revealing a clear bifurcation in the reaction pathway. FeN_4_ produced FFCA solely through the DFF intermediate and then stopped (Figure 2D), while CoN_4_ produced FFCA via both DFF and HMFCA, subsequently converting it to FDCA rapidly at low concentrations of the former (Figure 2E). Subsequently, probe experiments were conducted to elucidate this. The results demonstrated that FeN_4_ exhibited almost no catalytic activity toward the oxidation of HMFCA or FFCA (Figure 2F; Figure S13), confirming that it exclusively followed the HMF→DFF→FFCA pathway. In contrast, CoN_4_ exhibited catalytic activity toward DFF, HMFCA, and FFCA. This finding is consistent with the kinetic data, confirming the distinct product selectivity derived from HMF over the different catalysts.

Preliminary DFT calculations of the FFCA adsorption energies on the catalyst surfaces provided initial insights into this reactivity gap. As shown in Figure 2G, the adsorption energies of the key intermediates on the M‐N_4_ site were analyzed. For HMF and DFF, the adsorption energies on both catalysts were comparable, which is consistent with the experimental observation that both materials can effectively convert these substrates. In contrast, a significant divergence was observed in the adsorption energies of HMFCA (−0.15 vs. −0.28 eV) and FFCA (−0.14 vs. −0.29 eV) on the M‐N_4_ site. The notably weaker adsorption of these intermediates on FeN_4_ hinders their effective activation, making it difficult for the reaction to progress. This finding is consistent with experimental results showing that FeN_4_ does not follow the HMF→HMFCA→FFCA→FDCA pathway. Given that FeN_4_ does not utilize the HMFCA pathway and both catalysts can produce FFCA, the key selectivity difference resides in the fate of FFCA.

### 2.3 Electronic Mechanisms of Catalytic Selectivity

**FIGURE 3:**
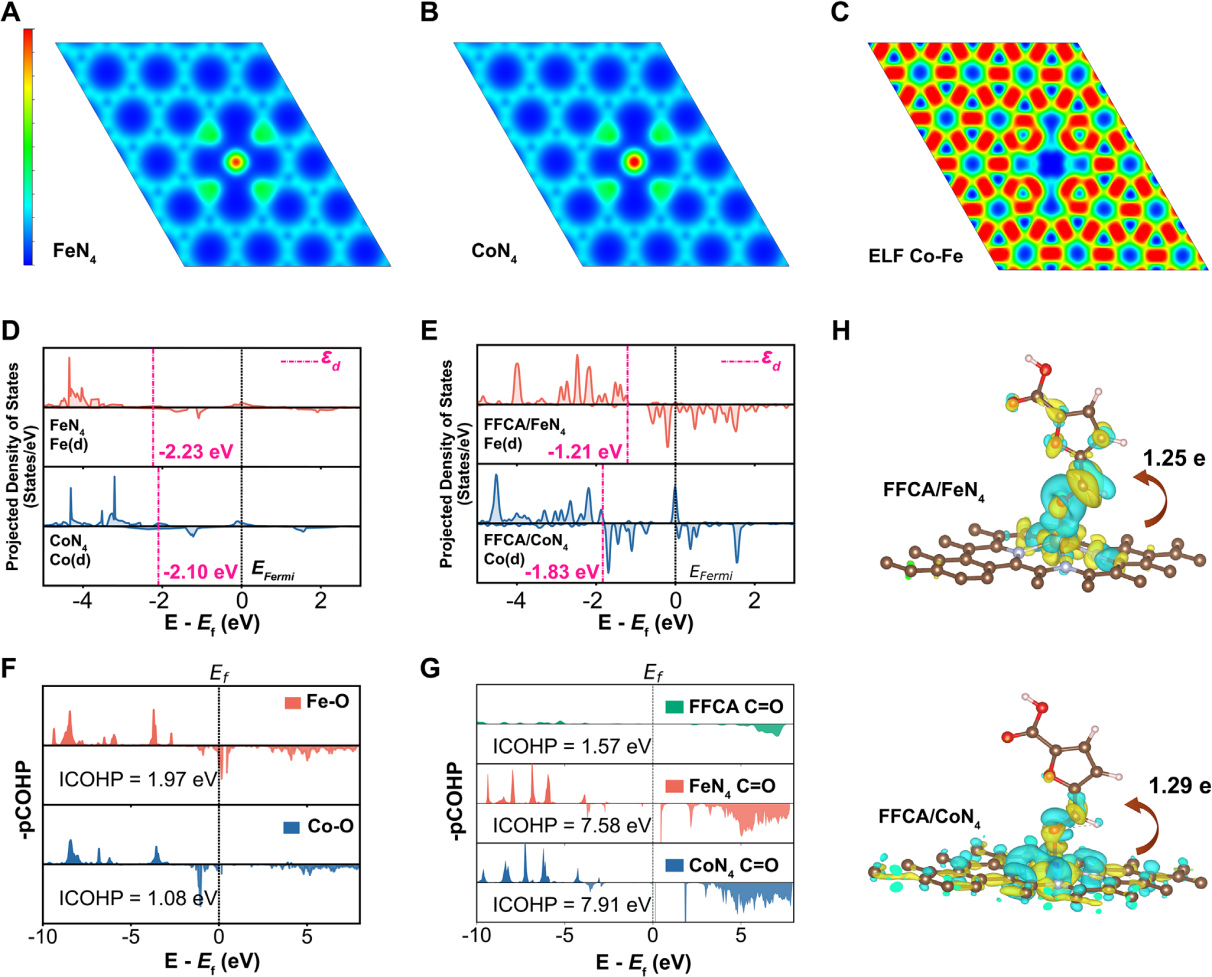
Theoretical analysis of the electronic origin of the catalytic selectivity. Charge‐density isosurfaces of (A) FeN_4_ and (B) CoN_4_ structures; (C) Electron Localization Function (ELF) plots for the pristine FeN_4_ and CoN_4_ structures; Projected density of states (PDOS) for the metal *d*‐orbitals of the pristine catalysts (D) before and (E) after FFCA adsorption, indicating the *d*‐band center shifts; (F) Projected Crystal Orbital Hamilton Population (pCOHP) analysis of the formed M─O bond and (G) the internal C═O bond of the FFCA adsorbate; (H) Differential charge density plots for FFCA adsorbed on FeN_4_ and CoN_4_, respectively.

To gain molecular‐level insight into the binding and activation mechanisms of FFCA on catalyst surfaces, comprehensive DFT calculations were performed to analyze the electronic interactions at the catalyst‐adsorbate interface. To assess the influence of the alkaline aqueous environment, including potential geminal‐diol pathways, preliminary calculations incorporating explicit water and hydroxide molecules were conducted (Figure S14). The computational results are consistent with the experimentally observed selectivity trends for FeN_4_ and CoN_4_ catalysts, and a detailed discussion of these findings will be presented in the section entitled “Role of Free Radicals in Reaction Selectivity ”. Given that the explicit solvation model yields selectivity trends in good agreement with experimental observations, thereby validating the reliability of our computational framework, subsequent mechanistic analyses were performed using the simplified gas‐phase model. This choice was made to prevent the excessive orbital complexity introduced by numerous solvent molecules from obscuring the intrinsic electronic interactions between the active site and the adsorbed intermediates. This simplified approach allows for a more precise characterization of the orbital hybridization (via pCOHP and DOS) between the metal *d*‐orbitals and the specific functional groups of the intermediates without interference from non‐bonding solvent molecules. First, the charge density isosurfaces of the FeN_4_ and CoN_4_ structures were obtained (Figure 3A,B). These results revealed a significant difference in their intrinsic electronic structures, which was further corroborated by the Electron Localization Function (ELF) plots (Figure 3C) [29]. Compared to FeN_4_, CoN_4_ exhibits enhanced electron localization, which is more favorable for FFCA adsorption, thereby facilitating its subsequent activation. The analysis of the *d*‐band center positions provides quantitative insight into these differences [30]. Upon FFCA adsorption, the *d*‐band center of the FeN_4_ site underwent a substantial upshift of 1.02 to −1.21 eV, whereas that of the CoN_4_ site shifted upward by only 0.27 to −1.83 eV (Figure 3D,E). Within the *d*‐band framework, a larger upshift reflects a stronger hybridization and more significant orbital coupling between the metal site and adsorbate. To dissect the nature of this coupling at the bond level, projected crystal orbital Hamiltonian population (pCOHP) analysis was employed to deconstruct the substrate‐binding interactions into contributions from individual chemical bonds [31]. A crucial distinction was revealed: the negative integrated COHP (‐ICOHP) for the local metal‐oxygen (M─O) bond formed between the catalyst and the aldehyde group of FFCA was 1.97 eV for Fe─O, significantly exceeding the 1.08 eV calculated for Co─O (Figure 3F,G). This demonstrates the formation of stronger, more covalent local bonds between the FeN_4_ sites and oxygen atoms of the adsorbate [32].

Paradoxically, this stronger local interaction is correlated with reduced catalytic activity in the oxidation pathway. The resolution of this paradox emerged from the analysis of the catalyst's effect on the intramolecular bonds within the FFCA. The pCOHP analysis focused on the intramolecular C═O bond of the aldehyde group, revealing higher ‐ICOHP values on the CoN_4_ surfaces, indicating more effective activation of the C═O bond. Consequently, although the Co─O coordination bond is weaker than the Fe─O bond, the CoN_4_ configuration activates the target C═O bond within the FFCA more effectively, thereby facilitating subsequent oxidation steps. Charge density difference (CDD) maps and Bader charge analysis (Figure 3H) further corroborated this finding. They demonstrated a substantially larger redistribution of electron density from the catalyst surface to the FFCA molecules on CoN_4_ compared to that on FeN_4_. This indicates that the electron cloud exchange on FeN_4_ is primarily localized between the metal and the molecule, whereas the entire substrate of CoN_4_ exhibits a stronger electronic exchange with the molecule. Meanwhile, the net electron transfer to the adsorbed FFCA on CoN_4_ reached approximately 1.29 e, which was higher than the 1.25 e transferred to FeN_4_. This enhanced charge transfer, which is consistent with the stronger overall adsorption energy on CoN_4_, signifies more profound polarization and electronic activation of the entire substrate molecule.

These computational results converge to provide a nuanced electronic picture that resolves the apparent contradiction between the local bond strength and catalytic activity. FeN_4_ sites engage with the FFCA through the formation of strong, localized, and highly covalent Fe─O bonds. Although this interaction effectively adsorbs the substrate molecule, it does not provide the electron redistribution required for the activation of the C═O bond. In contrast, the CoN_4_ site interacts with the FFCA molecule through polar covalent interactions, which are characterized by significant system‐level charge transfer. This delocalized electronic perturbation polarizes the entire adsorbate molecule, weakens the target intramolecular C═O bond, and renders it susceptible to oxidative attack, thereby promoting the oxidation of FFCA to FDCA.

### 2.4 Role of Free Radicals in Reaction Selectivity

**FIGURE 4:**
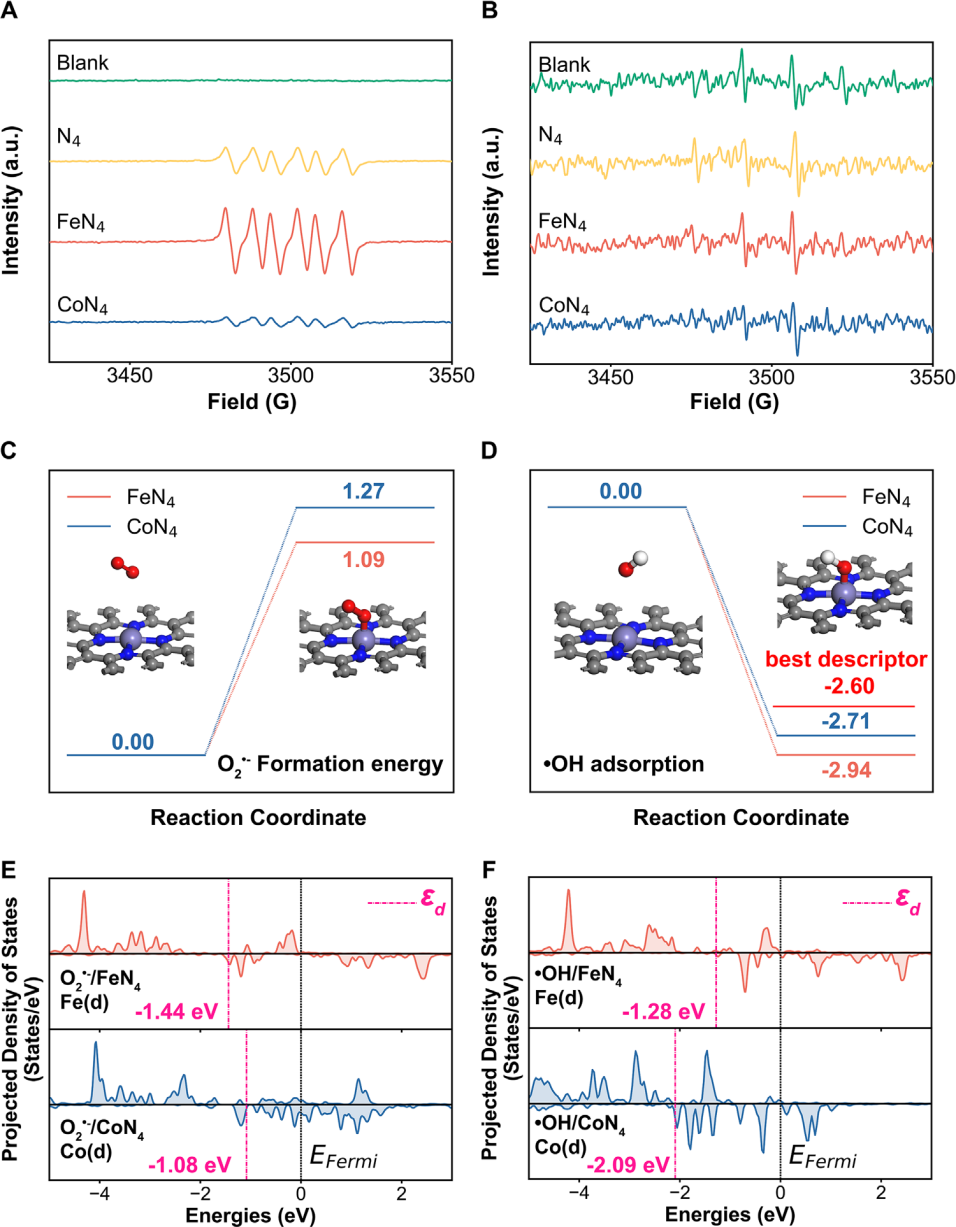
Analysis of catalyst interactions with reactive oxygen species (ROS). (A, B) Electron paramagnetic resonance (EPR) spectra for the detection of O_2_^•−^ and •OH radicals, respectively; (C) DFT‐calculated energy profile for O_2_^•−^ formation and (D) •OH adsorption on the FeN_4_ and CoN_4_ catalysts; (E,F) Projected density of states (PDOS) of the metal *d*‐orbitals upon adsorption of O_2_^•−^ and •OH, respectively, showing the resulting *d*‐band centers, N_4_ represents the metal‐free nitrogen‐doped carbon support.

It is widely known that reactive activation oxygen (ROS), notably hydroxyl radicals (•OH) or superoxide radicals (O_2_^•−^), are crucial in the oxidation of 5‐HMF [25]. Therefore, electron paramagnetic resonance (EPR) spectroscopy was employed to detect and identify the radical species in the SACs‐HMF co‐catalytic system, using DMPO as the spin‐trapping agent. The metal‐free nitrogen‐doped carbon support, synthesized using an identical procedure without the addition of metal precursors, is denoted as N_4_ and used as a control sample in the catalytic tests. Upon the addition of methanol to the reaction system, a characteristic DMPO/ O_2_^•−^ signal was observed for these catalysts. However, the signal was significantly stronger for FeN_4_ (Figure 4A). Notably, the detection of the 1:2:2:1 signal for the DMPO/•OH adduct in the catalyst‐free system indicates that HMF can spontaneously initiate ROS generation via aldehyde autoxidation [33]. This finding highlights the intrinsic activity of the formyl group in HMF to trigger radical chains without catalyst mediation (Figure 4B). Following catalyst addition, the positions of the DMPO/•OH signals remained unchanged in all the reaction systems. However, a marked decrease in the signal intensity was observed for CoN_4_, suggesting the critical role of •OH in the CoN_4_ catalytic system [34]. To gain deeper insight into the reaction energy barriers, explicit DFT models incorporating all stoichiometrically relevant oxygen and water species were constructed to simulate the catalytic cycle under aqueous‐phase conditions (Figure S14). Starting from the co‐adsorption of O_2_ and HMF on the MN_4_ sites (Steps 1–2), molecular oxygen is activated to form the OOH intermediate (Step 3), which serves as the key reactive oxygen species for aldehyde C─H bond activation. The subsequent oxidation of HMF to DFF and further to FFCA proceeds through coupled proton‐transfer and oxidation steps, generating H_2_O_2_ and H_2_O as co‐adsorbed intermediates at the catalyst surface (Steps 5–8). This pathway captures the essential role of proton and oxygen transfer in the aqueous‐phase aldehyde‐to‐acid conversion.

To further investigate the effect of ROS on the selective oxidation of HMF, the adsorption and generation energetics of different free radical on the M‐N_4_ site were calculated using DFT. Figure 4C illustrates the generation of energy of O_2_^•−^ at the M‐N_4_ site. The results show that FeN_4_ exhibits a lower activation barrier (1.09 eV) for oxygen than CoN_4_ (1.27 eV), suggesting a stronger tendency to activate oxygen for O_2_^•−^ generation. In contrast, the adsorption of •OH on the M‐N_4_ sites more closely follows the Sabatier principle, which necessitates a moderate binding strength (neither too high nor too low) for the reaction to proceed efficiently. As shown in Figure 4D, FeN_4_ exhibits strong •OH binding at −2.94 eV, which leads to site poisoning and impedes catalysis, whereas CoN_4_, with a more moderate binding energy of −2.71 eV, approaches the optimal window (−2.60 eV) for •OH turnover, thereby facilitating the reaction process [35].

Subsequent projected density of states (PDOS) and corresponding *d*‐band center (*ε_d_*) analyses provided strong evidence regarding the bonding mechanism to explain the differential behavior of free radicals. The adsorption of O_2_^•−^ onto the M‐N_4_ site induces hybridization between the Fe *d*‐orbitals and the superoxide *p*‐orbitals owing to its electron‐nucleophilic character, which is corroborated by the upshift of the FeN_4_ *d*‐band center from −2.23 to −1.44 eV (Figure 4E). In contrast, the interaction between CoN_4_ and O_2_^•−^ manifests both strong orbital hybridization and charge transfer, which is discernible from the overlap between the Co *d*‐orbitals and the π^*^ orbitals of O_2_^•−^. These findings suggest that the stronger chemical affinity of O_2_^•−^ for CoN_4_ sites increases the energy barrier for its formation by overly stabilizing the reactant state. This mechanistic insight explains why CoN_4_ is less capable of generating O_2_^•−^ than FeN_4_. In the •OH adsorption model (Figure 4F), the *d*‐band center of FeN_4_ shifted significantly from −2.23 to −1.28 eV. This pronounced upshift originates from the strong *d*‐orbital‐driven covalent bonding, which is consistent with the strong adsorption of •OH on FeN_4_. In contrast, the adsorption of •OH does not induce noticeable changes in the *d*‐band electronic structure of CoN_4_ (despite the considerable adsorption energy between them), revealing a remarkable decoupling between the energetics and *d*‐orbital electronic properties. This phenomenon may be related to the *d*‐electron configuration of CoN_4_, where the additional *d*‐electron occupying the *d_z_^2^* orbital may generate Pauli repulsion against •OH, thereby influencing its adsorption [30].

**FIGURE 5:**
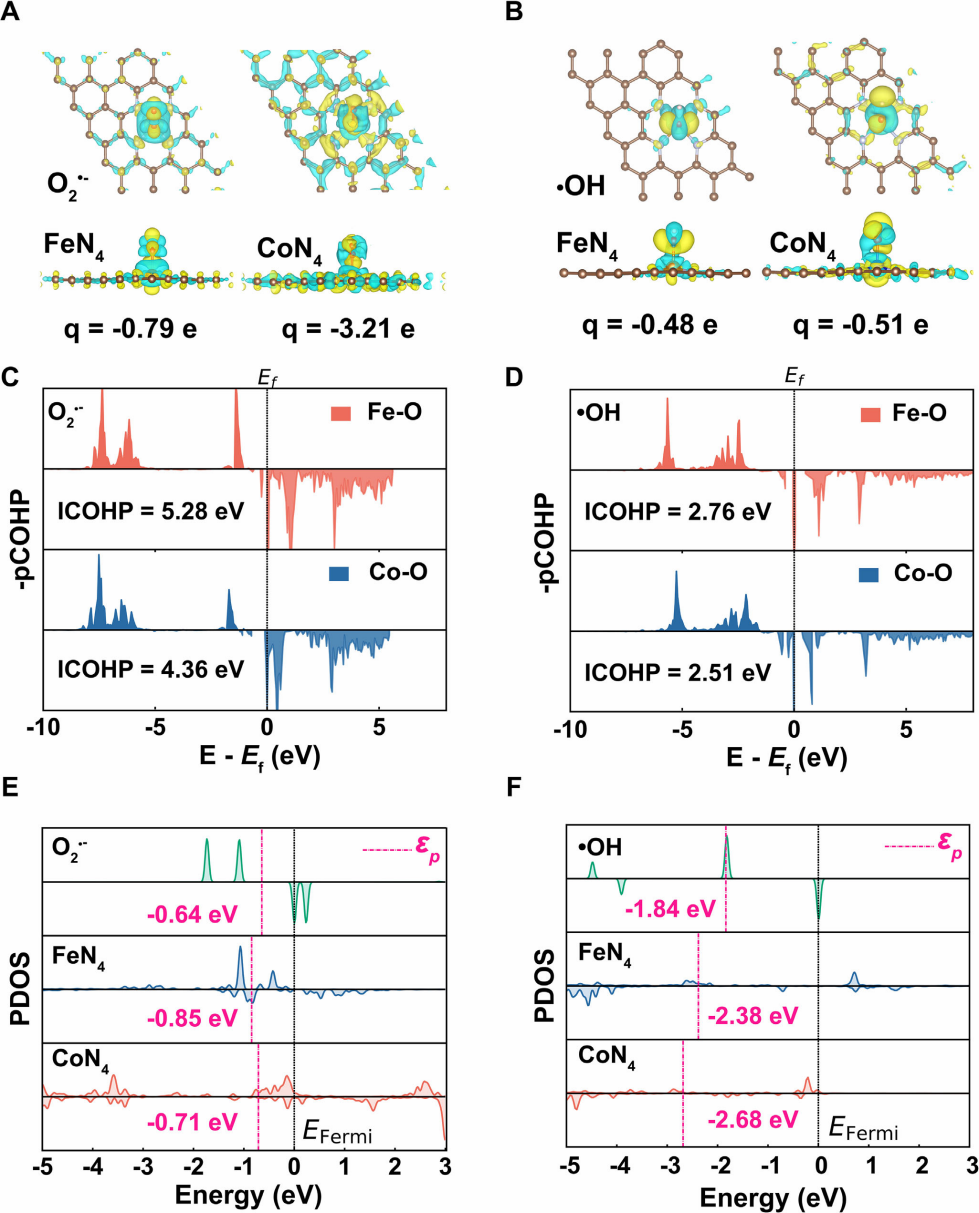
Electronic structure analysis of adsorbed reactive oxygen species. (A,B) Differential charge density plots for O_2_^•−^ and •OH adsorbed on the FeN_4_ and CoN_4_ surfaces; (C,D) corresponding ‐pCOHP analysis of the M─O covalent bond for the adsorbed O_2_^•−^ and •OH species; (E,F) projected density of states (PDOS) of the oxygen *p*‐orbitals for adsorbed O_2_^•−^ and •OH, indicating the position of the O *p*‐band center (*ε_p_*).

Although the aforementioned results provide explanations from the perspectives of energetics and electronic orbital theory, respectively, the electron transfer behavior governing the interactions between free radicals and active sites can offer a more precise interpretation of the observed selectivity. The results indicate that the charge transfer between O_2_^•−^ and FeN_4_ is only ‐0.79 e, while that with CoN_4_ reaches −3.21 e (Figure 5A). In the •OH adsorption model, although the two catalysts exhibited notable differences in the *d*‐band center shifts, their charge transfer amounts showed no significant variation, both demonstrating relatively weak charge exchange (Figure 5B). This pronounced difference is consistent with the adsorption behavior of the radicals on the two types of catalyst surfaces. The underlying origin lies in the distinct charge transfer pathways: in CoN_4_, charge is delocalized from the metal center to the entire support surface, resulting in global electron exchange, whereas in FeN_4_, charge transfer remains predominantly localized near the metal active site. Furthermore, the strength of the M─O bonds between the free radical and catalyst was analyzed using the ‐ICOHP metric. The results indicate that the Fe─O bond is stronger than the Co─O bond in both adsorption models, as evidenced by the higher ‐ICOHP values (Figure 5C,D). This not only provides direct evidence for the upward shift of the *d*‐band center in FeN_4_ due to stronger bonding but also elucidates the decoupling phenomenon in CoN_4_. By employing oxygen *p*‐band analysis, the study was further extended to adsorbed radicals. The results show that the oxygen *p*‐states of O_2_^•−^ are closer to the Fermi level on CoN_4_ (Figure 5E), whereas those of •OH are closer to FeN_4_ (Figure 5F). The Fe─O bond exhibits a significantly higher integrated COHP (‐ICOHP) value, which signifies a stronger degree of covalency and a more robust orbital overlap between the Fe 3d and O 2p states. This intensified covalency is the primary electronic origin of the more negative adsorption energy observed for FeN_4_. Conversely, the Co─O bond displays a lower ‐ICOHP value, indicating a weaker covalent interaction that is consistent with its more moderate adsorption energy. This bonding characteristic, further supported by the observed charge transfer and *d*‐band center shifts, provides a unified explanation for the site‐dependent adsorption strengths and the resulting catalytic selectivity. In summary, the electronic structure analyses, including charge transfer pathways, *d*‐band center shifts, ‐ICOHP values, and oxygen *p*‐band positions, collectively reveal a consistent result. The strong, localized Fe‐O covalent interaction on FeN_4_ effectively stabilizes and activates O_2_^•−^, thereby promoting HMF oxidation. In contrast, the weaker, delocalized bonding on CoN_4_ favors the preferential consumption of •OH radicals in this process.

**SCHEME 1:**
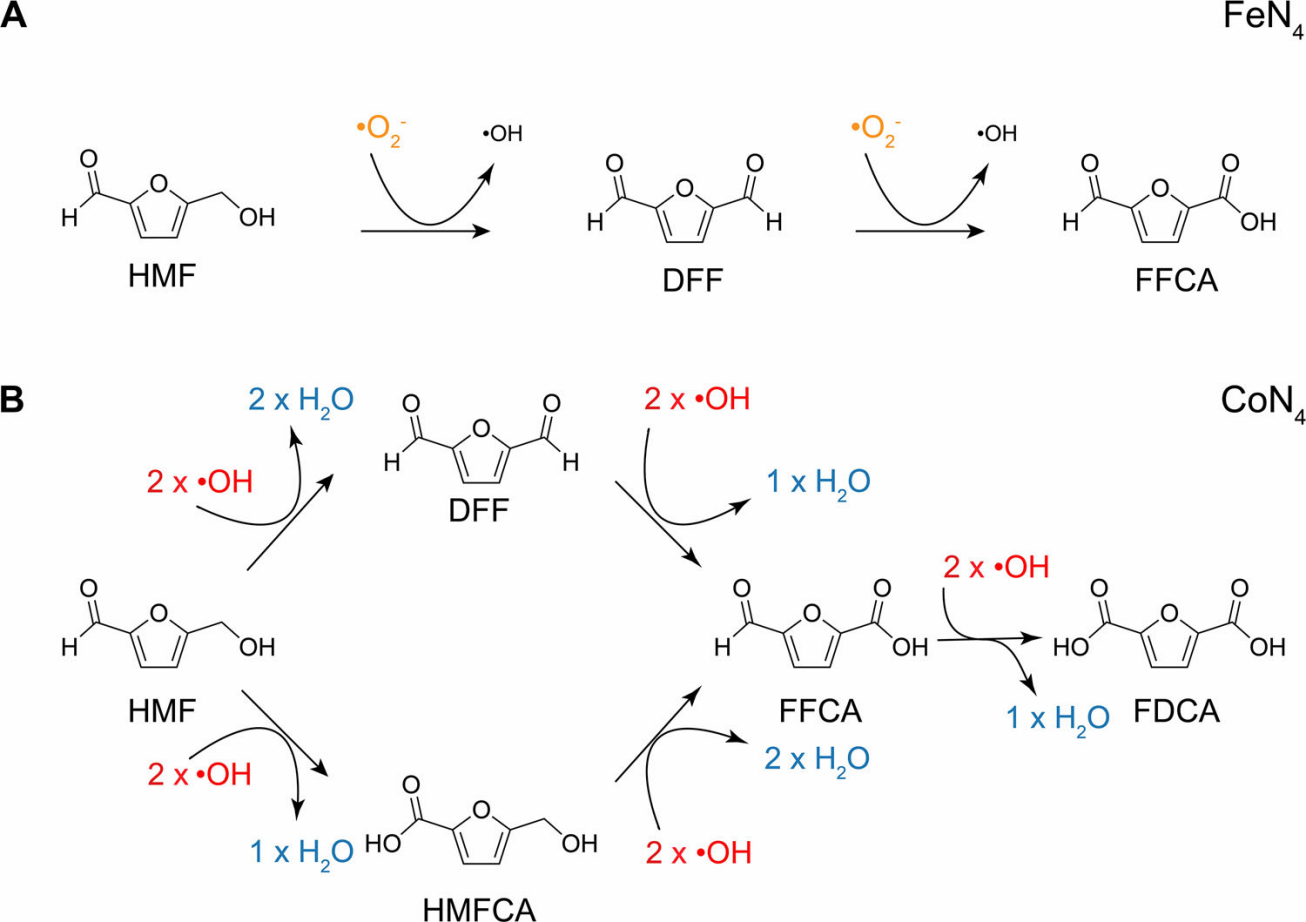
(A) Reaction pathway for the oxidation of HMF to FFCA over the FeN_4_ catalyst, proceeding through a DFF intermediate with superoxide radical (O_2_^•−^) mediation. (B) Parallel reaction pathways for the complete oxidation of HMF to FDCA over the CoN_4_ catalyst, proceeding either through the DFF intermediate (upper route) or through the HMFCA intermediate (lower route).

Based on the combined experimental and DFT results, a potential catalytic pathway for the selective oxidation of HMF was proposed. As shown in Scheme 1, FeN_4_ mediates the oxidation of HMF along the HMF→DFF→FFCA pathway using superoxide anions as oxidants. Although this pathway is thermodynamically favorable (Figure S15), the subsequent oxidation of FFCA is kinetically hindered by a high energy barrier, resulting in selective termination at FFCA. In contrast, CoN_4_ primarily mediates HMF oxidation via •OH radicals, a process that occurs through two distinct pathways (Scheme 1). Both pathways are thermodynamically spontaneous and encounter relatively low kinetic barriers. Consequently, the reaction can proceed along either route, leading to a product selectivity profile distinct from that of FeN_4_.

### 2.5 Cycling Stability Assessment

**FIGURE 6:**
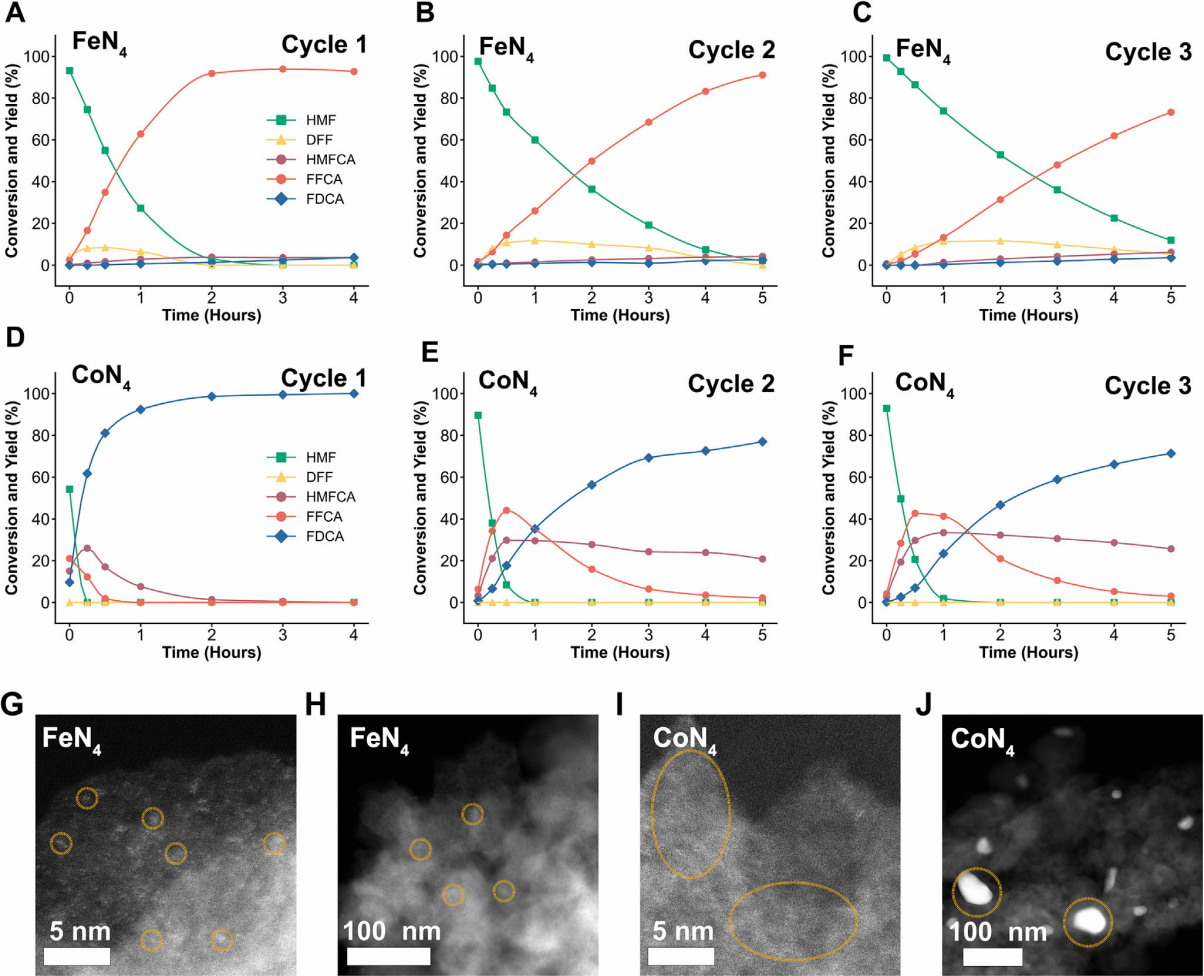
Catalyst stability and reusability evaluation. (A–C) Time‐course profiles for HMF oxidation over the FeN_4_ catalyst in three consecutive cycles. (D–F) Corresponding recycling tests for the CoN_4_ catalyst, showing progressive deactivation. (G,H) HAADF‐STEM images of the FeN_4_ catalyst after stability testing, confirming its atomic dispersion. (I,J) HAADF‐STEM images of the used CoN_4_ catalyst, revealing the aggregation of Co single atoms into nanoparticles.

Long‐term operational stability is crucial for practical catalytic applications. Therefore, the stability of the catalyst was assessed through systematic recycling experiments. The kinetic profiles of FeN_4_ for three consecutive runs exhibited substantial overlap (Figure 6A,C), with HMF conversion and FFCA selectivity stabilizing at approximately 80% after 5 h, indicating good recyclability with moderate activity retention. In contrast, CoN_4_ underwent severe deactivation during the recycling studies. In the initial cycle (Figure 6D), CoN_4_ exhibited high catalytic activity, achieving complete HMF to FDCA conversion within 1 h with near‐100% selectivity. However, the catalytic performance deteriorated markedly in subsequent cycles (Figure 6E,F), with the HMF conversion declining and the FDCA yield decreasing to 75% by the third cycle. Concomitantly, DFF and FFCA intermediates accumulated, indicating a compromised deep oxidation capability. The STEM images (Figure 6G,H) of the post‐reaction catalysts confirmed the atomically dispersed iron active sites and intact single‐atom configuration in FeN_4_. However, the irregular Co nanoparticles in the CoN_4_ carbon matrix were identified as the primary reason for its inferior catalytic performance and selectivity (Figure 6I,J). The difference in stability is likely related to their respective reaction pathways and coordination environments. The ICP‐OES analysis results of the post‐reaction filtrates are summarized in Table S5. The dissolved Fe content from FeN_4_ was approximately 245 ppb, whereas only a trace amount of Co leaching was detected from CoN_4_ (approximately 10 ppb), with the extent of metal dissolution decreasing progressively with successive catalytic cycles. To evaluate the potential contribution of leached species to the catalytic performance, the filtrate obtained from the FeN_4_ system was subjected to a hot filtration test, as shown in Figure S16. The results indicate that the catalytic effect of dissolved Fe^2+^/Fe^3+^ species on HMF conversion is negligible, confirming the heterogeneous nature of the catalysis. The progressive CoN_4_ deactivation is attributed to the combined effects of single‐atom aggregation, minor metal leaching, and possible organic deposition (humin‐type species) on the catalyst surface [14]. The recycling experiments demonstrated that FeN_4_ maintained stable catalytic performance over three consecutive cycles with well‐preserved single‐atom dispersion, whereas CoN_4_ suffered from progressive deactivation accompanied by nanoparticle aggregation. This contrasting stability can be rationalized by the distinct reactive oxygen species involved and the intrinsic coordination chemistry of the two catalysts. The selective oxidation of HMF over FeN_4_ is mediated by the relatively mild O_2_^•−^, which alleviates local oxidative stress on the active sites and thereby enhances long‐term durability. In contrast, the highly oxidizing •OH generated at CoN_4_ sites imposes an aggressive chemical environment that accelerates the destabilization of the metal centers and promotes single‐atom aggregation. Moreover, the Fe─N bond possesses a higher bond dissociation energy than the Co─N bond, which further reinforces the structural integrity of FeN_4_ under prolonged oxidative conditions.

## 3 Conclusion

In this study, precise control over the selective oxidation products of HMF was achieved solely by altering the metal centers in M‐N‐C SACs. Specifically, FeN_4_ achieved a 93.9% selectivity toward FFCA at 99.9% HMF conversion, whereas CoN_4_, under the same conversion level, enabled near‐complete formation of FDCA. This divergence in selectivity is closely related to the electronic structure of the metal sites in the catalysts, which governs the adsorption and activation processes of the key intermediate, FFCA. Furthermore, such differences in the electronic structure can selectively modulate the generation and consumption of radical species. For instance, FeN_4_ preferentially generates O_2_^•−^ to achieve the partial oxidation of HMF, whereas CoN_4_ promotes the consumption of •OH to accelerate the final oxidation step. Notably, this radical selectivity also influences catalyst stability: the highly oxidative •OH causes CoN_4_ to agglomerate and deactivate, whereas the relatively mild O_2_^•−^ does not significantly alter the catalytic activity or selectivity of FeN_4_. In summary, this study presents an efficient strategy for the controllable oxidation of HMF and provides important theoretical insights into tailoring selectivity through the intrinsic, metal‐center‐dependent electronic structure of single‐atom catalysts.

## Conflicts of Interest

The authors declare no conflicts of interest.

## Supporting information




**Supporting File**: advs75374‐sup‐0001‐SuppMat.docx.

## Data Availability

The data that support the findings of this study are available in the supplementary material of this article.
