# Supplementary material for: MetalCenter‐Dependent Selectivity Divergence in MN4 Single‐Atom Catalysts for Aerobic HMF Oxidation
Source: Adv Sci (Weinh). 2026 Apr 21;13(40):e24182. doi: 10.1002/advs.202524182 (PMC13335703; doi:10.1002/advs.202524182)
Supplement: Supplementary file 1 — Supporting File: advs75374‐sup‐0001‐SuppMat.docx. [file ADVS-13-e24182-s001.docx]

**Supplementary Information**

**Metal-Center-Dependent Selectivity Divergence in MN_4_ Single-Atom Catalysts for Aerobic HMF Oxidation**

*Haoyu Wang ^a^, Zhilong Ye ^a^, Weihao Wang ^a^，Zhi Hu ^b^，Qing Tian ^c*^, Jie Gao^d, a*^, Lianbing Zhang ^a*^*

^a^ School of Life Science and Technology, Northwestern Polytechnical University, Xi'an 710072, China

^b^ Zhejiang Key Laboratory of Low-carbon Control Technology for Industrial Pollution, College of Environment, Zhejiang University of Technology, Hangzhou 310032, China

^c^ Nanozyme Laboratory in Zhongyuan, Zhengzhou, 451100, China

^d^ Nanozyme Laboratory in Zhongyuan, College of Chemistry and Molecular Sciences, Henan University, Zhengzhou 450000, Henan, PR China

^*^ Corresponding author, E-mail: [tianqing@nanozyme.tech](mailto:tianqing@nanozyme.tech), [jgao@nwpu.edu.cn](mailto:jgao@nwpu.edu.cn), [lbzhang@nwpu.edu.cn](mailto:lbzhang@nwpu.edu.cn)

**Supplementary notes**

**1. Methods**

The microstructural and morphological characteristics of the FeN_4_ and CoN_4_ catalysts were examined using double Cs-corrector transmission electron microscopy (Themis Z, Thermo Fisher Scientific) at an acceleration voltage of 300 kV and high-resolution transmission electron microscopy (TEM, FEI Talos F200X, Thermo Fisher Scientific) at an acceleration voltage of 200 kV. X-ray diffraction (XRD) patterns were acquired using a Smart Lab SE instrument (Rigaku) with Cu K*α* radiation over a 2*θ* range of 5-90° at a scan rate of 2^o^/min. The surface chemical states and compositions of the catalysts were analyzed using X-ray photoelectron spectroscopy (XPS, K-Alpha, Thermo Fisher Scientific) with Al K*α* radiation (hν = 1486.6 eV). X-ray absorption fine structure (XAFS) spectra were collected at beamline BL14W1 of the Shanghai Synchrotron Radiation Facility and analyzed using Athena and Artemis modules in the IFEFFIT software package. The metal content was determined by inductively coupled plasma optical emission spectrometry (ICP-OES, 5110, Agilent) after the acid digestion of the catalyst samples. The textural properties, including the specific surface area, pore volume, and pore size distribution, were evaluated from N_2_ adsorption-desorption isotherms measured at 77 K (3H-2000PS2, BSD INSTRUMENT). The specific surface area was calculated using the Brunauer-Emmett-Teller (BET) method, and the pore size distribution was derived from the desorption branch using the non-local Density Functional Theory (NLDFT) method. Electron paramagnetic resonance (EPR) spectra were recorded at ambient temperature on a Bruker EMX-plus spectrometer operating at the X-band frequency (~9.4 GHz), and the instrumental parameters, including the microwave power and modulation amplitude, were set to 2 mW (microwave power) and 1 G (modulation amplitude), optimized using the spectrometer’s standard tuning functions to maximize the signal-to-noise ratio for radical adduct signals (g ≈ 2).

**2. Materials and Reagents**

Zinc nitrate hexahydrate (Zn(NO_3_)_2_·6H_2_O, CP), sodium hydroxide (NaOH, AR), iron(II) sulfate heptahydrate (FeSO_4_·7H_2_O, AR), and methanol anhydrous (CH_3_OH, AR) were obtained from Sinopharm Chemical Reagent Co., Ltd. Cobalt nitrate hexahydrate (Co(NO_3_)_2_·6H_2_O, AR, 99%), ammonium formate (CH_5_NO_2_, AR, ≥99.0%), and 4-Amino-2,2,6,6-tetramethylpiperidine (C_9_H_20_N_2_, >98.0%) were obtained from Shanghai Aladdin Bio-Chem Technology Co., Ltd. 5,5-Dimethyl-1-pyrroline N-oxide (C_6_H_11_NO, 97%) was obtained from Macklin. Methanol for the liquid chromatography mobile phase (CH_4_O, HPLC Grade) was obtained from PUREDiL Chemicals. Water was purified using a Merck Milli-Q system.

**3. Synthesis of FeN_4_**

FeSO_4_·7H_2_O (0.2419 g) and Zn(NO_3_)_2_·6H_2_O (8.9247 g) were dissolved in 500 mL of methanol to prepare Solution A. Separately, dimethylimidazole (19.700 g) was dissolved in 400 mL of methanol to prepare Solution B. Solution B was then added to Solution A under vigorous stirring at 1200 rpm at room temperature (20 °C). After the reaction, the resulting precipitate was collected by centrifugation at 12,000 rpm for 5 minutes at room temperature. The collected solid was washed three times with anhydrous methanol and dried overnight at 80 °C. The dried precursor was thoroughly ground, placed into a porcelain boat, and transferred to a tube furnace. Under a continuous nitrogen flow of 50 mL/min, the sample was heated to 1000 °C at a ramp rate of 5 °C/min, followed by natural cooling to room temperature. Finally, the calcined product was ground to yield the target material.

**4. Synthesis of CoN_4_**

CoNO_3_·6H_2_O (0.2532 g) and Zn(NO_3_)_2_·6H_2_O were dissolved in 500 mL of methanol to prepare Solution A. Separately, dimethylimidazole (19.700 g) was dissolved in 400 mL of methanol to prepare Solution B. Solution B was then added to Solution A under vigorous stirring at 1200 rpm at room temperature (20 °C). After the reaction, the resulting precipitate was collected by centrifugation at 12,000 rpm for 5 minutes at room temperature. The collected solid was washed three times with anhydrous methanol and dried overnight at 80 °C. The dried precursor was thoroughly ground, placed into a porcelain boat, and transferred to a tube furnace. Under a continuous nitrogen flow of 50 mL/min, the sample was heated to 1000 °C at a ramp rate of 5 °C/min, followed by natural cooling to room temperature. Finally, the calcined product was ground to yield the target material.

**5. Synthesis of N_4_**

Zn(NO_3_)_2_·6H_2_O (8.9247 g) were dissolved in 500 mL of methanol to prepare Solution A. Separately, dimethylimidazole (19.700 g) was dissolved in 400 mL of methanol to prepare Solution B. Solution B was then added to Solution A under vigorous stirring at 1200 rpm at room temperature (20 °C). After the reaction, the resulting precipitate was collected by centrifugation at 12,000 rpm for 5 minutes at room temperature. The collected solid was washed three times with anhydrous methanol and dried overnight at 80 °C. The dried precursor was thoroughly ground, placed into a crucible, and transferred to a porcelain boat. Under a continuous nitrogen flow of 50 mL/min, the sample was heated to 1000 °C at a ramp rate of 5 °C/min, followed by natural cooling to room temperature. Finally, the calcined product was ground to yield the target material.

**6. Catalytic Activity Tests**

The catalytic reactions were conducted in a 250 mL three-neck round-bottom flask. In a typical procedure, 5 mmol of NaOH, 0.5 mmol of HMF, 40 mg of catalyst, and 100 mL of H_2_O were added to the reactor, medical grade oxygen was introduced at a flow rate of 50 mL min^-1^, and the mixture was stirred at 1200 rpm at 60 °C for 3 h. After the reaction, the catalyst was recovered by filtration. For the recycling experiments, the heterogeneous catalyst was separated by filtration, washed sequentially with acetonitrile, methanol, and deionized water (five times each), and dried in an oven at 90 °C for 12 h before the next cycle.

**7. HPLC Analysis**

High-performance liquid chromatography (HPLC) coupled with a UV-vis detector was used to analyze the concentrations of organic compounds in the electrolyte. The detection wavelength for HMF, HMFCA, DFF, FFCA, and FDCA was 265 nm. The mobile phase was composed of methanol and a 5 mM ammonium formate aqueous solution in a volume ratio of 15:85, at a flow rate of 0.4 mL/min. The column was maintained at a constant temperature of 40°C. A 4.6 mm × 250 mm Elite Supersil AQ-C18 column was used. The separation time was 25 min for all samples. HMF and its oxidation products were calculated according to the calibration curves of standard compounds with known concentrations (Figure S7).

**8. Detection of Free Radical**

5,5-dimethyl-1-pyrroline N-oxide (DMPO, 97%) was purchased from Macklin and used as received. 5-Hydroxymethylfurfural (HMF), methanol (MeOH), and deionized (DI) water were used in the experiments. Stock suspensions of the FeN_4_ and CoN_4_ catalysts were prepared separately by dispersing 4 mg of each material in 1 mL DI water. The suspensions were ultrasonicated prior to use to ensure homogeneity. A 10 mM stock solution of HMF was prepared using DI water. EPR spectra were recorded using a Bruker EMX-plus spectrometer at ambient temperature.

8.1. Detection of Hydroxyl Radicals (•OH)

In a typical experiment, 750 µL of DI water was added to a 2 mL Eppendorf tube and placed in a metal block heater maintained at 60^o^C. After 5 min of preheating, 100 µL of the respective catalyst suspension (4 mg/mL) and 50 µL of DMPO were added sequentially. The mixture was continuously purged with medical-grade O_2_ gas at a flow rate of 50 mL/min. The reaction was initiated by adding 100 µL of a 10 mM HMF solution. After incubation for 5 min at 60 °C, the solution was transferred to a capillary tube, and the EPR spectrum of the DMPO-•OH product was immediately recorded. This procedure was followed for both FeN_4_ and CoN_4_ catalysts.

8.2. Detection of Superoxide Radicals (O_2_•⁻)

The procedure for detecting superoxide radicals was nearly identical to that for •OH detection, with a key modification. Methanol was used as the reaction solvent, instead of deionized water. Methanol acts as an efficient quencher of hydroxyl radicals, thereby enabling the selective detection of the DMPO-O_2_•⁻ adduct in the system.

1. **Optimization of the Reaction Conditions**

To identify the optimal conditions for the catalytic conversion of HMF, the effects of the reaction temperature and initial pH were systematically investigated. First, the influence of temperature was examined at a fixed initial pH of 12.5 (Figure 2B in the main text). While the HMF conversion rate generally increased with temperature, the carbon balance began to decline at temperatures above 60 °C, likely due to the promotion of side reactions. Therefore, 60 °C was selected as the optimal temperature to balance the high conversion with excellent product selectivity.

Next, the effect of the initial pH was explored while maintaining the temperature at 60 °C (Figure S8). The HMF conversion rate increased with alkalinity. However, a sharp decrease in the carbon balance was noted when the pH was raised above 12.5, indicating the probable degradation of the substrate or products under stronger basic conditions. Consequently, an initial pH of 12.5 was determined to be the optimal value. Control experiments were performed without any catalyst (Figure S9) confirmed that although the substrate undergoes some conversion on its own under these thermal and alkaline conditions, the presence of our catalyst is crucial for achieving high efficiency. In summary, the optimal conditions for the reaction were 60 °C and an initial pH of 12.5.

**10. Determination of HMF and Oxidation Products**

The HMF conversion, product yields, and product selectivity were calculated using the following equations:

$$\text{HMF Conversion}(\%)\text{=}\frac{\text{(Initial HMF concentration - Final HMF concentration)}}{\text{Initial HMF concentration}}\times100$$

$$\text{Product Yield}(\%)\text{=}\frac{\text{Moles of product formed}}{\text{Moles of initial HMF}}\times100$$

$$\text{Selectivity to Product}(\%)\text{=}\frac{\text{Yield of specific product}}{\text{HMF Conversion}}\times100$$

1. **Turnover Frequency**

The number of active sites was based on metal loading from ICP-OES (e.g., 0.99 wt.% Fe for FeN_4_, assuming all metal atoms are active single-atom sites). Based on initial rate measurements (<20% conversion), the TOF for FeN_4_ was determined to be 18.12 h⁻¹ and for CoN_4_ was 18.42 h⁻¹.

The turnover frequency was calculated using the following equation:

$$\text{TOF }(h^{-1})=\frac{\text{Moles of HMF converted}}{\text{Moles of metal atoms in catalyst}\times\text{Reaction time in hours}}$$

1. **Activation Energy (E_a_) from Arrhenius Plots**

The reactions were conducted at different temperatures under otherwise identical conditions. The initial rates (r) were measured as the slope of the HMF concentration versus time in the linear regime (<20% conversion).

Rate Constant (k): Assuming pseudo-first-order kinetics for HMF (excess O_2_)

Arrhenius Equation:

$$k=A\exp\left(-\frac{E_{a}}{RT}\right)$$

where A is the pre-exponential factor, R is the gas constant (8.314 J mol^-1^ K^-1^), and T is the temperature in Kelvin.

Plot: ln(k) vs. 1/T (Arrhenius plot); Ea = -slope × R

Fitting: Linear regression using software (e.g., matplot)

**13. Computation details**

All spin-polarized density functional theory (DFT) calculations were performed using the Vienna Ab initio Simulation Package (VASP) code. The projector-augmented wave (PAW) method was employed to describe the interactions between the ion cores and valence electrons. The exchange-correlation effects were described using the Perdew-Burke-Ernzerhof (PBE) functional within the generalized gradient approximation (GGA). A plane-wave basis set with a kinetic energy cutoff of 520 eV was used in all calculations.

The electronic structure was relaxed until the total energy difference between successive self-consistent field (SCF) cycles was less than 10^-5^ eV. The geometries of all structures were fully optimized using a conjugate gradient algorithm until the forces on each atom were below 0.02 eV/Å. A Gaussian smearing approach with a smearing width of 0.05 eV was applied to electronic occupations. For structural relaxation, the ionic positions and cell shapes were varied accordingly. To account for the strong on-site Coulomb interactions of specific d-electrons, the GGA+U method was utilized, with the magnetic moments of the atoms carefully initialized.

To bridge the gap between the theoretical models and the experimental reaction medium (strongly alkaline aqueous solution), the following modeling strategies were adopted:

**13.1 Reaction Medium & Mechanism:** For the investigation of the reaction coordinates, particularly the aldehyde-to-acid transformation which proceeds through a geminal-diol intermediate in basic media, preliminary validations were conducted using an explicit model incorporating water molecules (H_2_O) and hydroxide ions (OH^-^) (**see Figure S15**). These tests confirmed that the inclusion of explicit solvation and base-assisted chemistry did not alter the fundamental energy barrier trends between the FeN_4_ and CoN_4_ active sites.

**13.2 Simplified Models for Electronic Analysis:** For the deconstruction of chemical bonding and orbital interactions, simplified gas-phase-like models (focusing on the MN_4_ site and the adsorbate) were utilized for the Projected Crystal Orbital Hamilton Population (pCOHP) and Density of States (DOS) analyses. This approach was chosen to maximize the precision of local electronic descriptors and avoid numerical noise from non-bonding solvent molecules, ensuring a clear interpretation of the orbital hybridization between the metal centers and the key intermediates.

Chemical bonding and orbital interactions were deconstructed using the LOBSTER program. Structural models, including surface slab generation and data preprocessing, were facilitated by the Materials Project Python library.

**14. Heterogeneity and Leaching Analysis**

A typical reaction was carried out in a reaction vessel with a total liquid volume of 10 mL. First, 5 mL of the target leaching solution was collected and transferred into the reactor. Separately, a substrate solution was prepared by dissolving 0.25 mmol of 5-hydroxymethylfurfural (HMF) and 0.25 mmol of NaOH in 5 mL of deionized water. This substrate solution was then added to the leaching solution in the reactor, yielding a 10 mL reaction mixture. The reaction system was heated to 60 °C under ambient pressure. Oxygen gas (O_2_) was continuously bubbled through the reaction mixture at a constant flow rate of 50 mL/min. To monitor the reaction progress, aliquots were taken from the reaction mixture at designated time intervals (0, 15, 30, 45, and 60 min). The samples were appropriately processed and analyzed by High-Performance Liquid Chromatography (HPLC). The baseline of the resulting chromatograms was background-subtracted to eliminate the interference of dissolved ion signals.

**Supplementary figures**


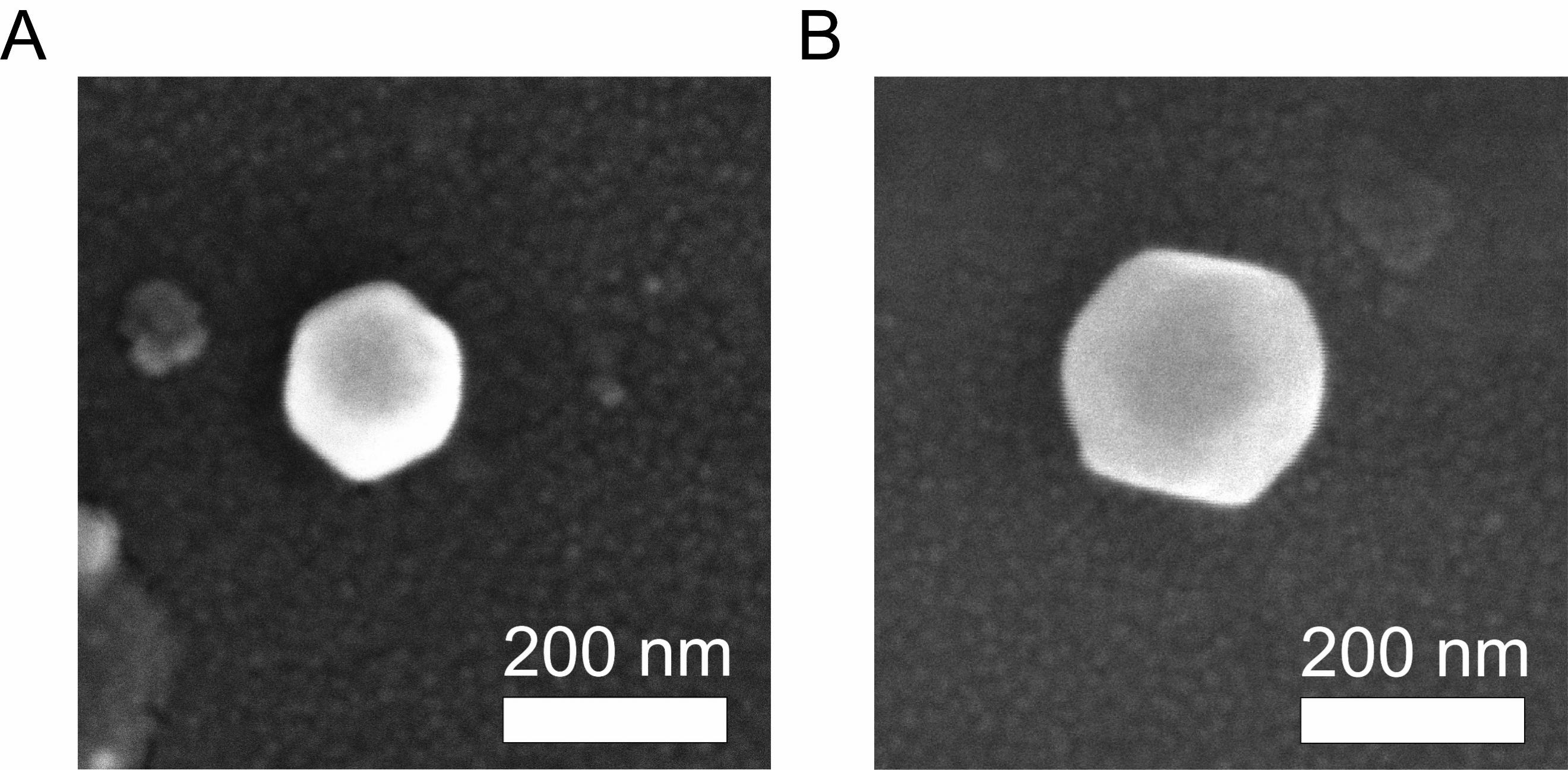


Figure S1 Scanning electron microscopy (SEM) images of the synthesized zeolitic imidazolate framework (ZIF) precursors. (A) Morphology of the Fe-based Zeolitic Imidazolate Framework (Fe-ZIF) precursor. (B) Morphology of the Cobalt-based Zeolitic Imidazolate Framework (Co-ZIF) precursor. The scale bars are indicated at the bottom right of each image.


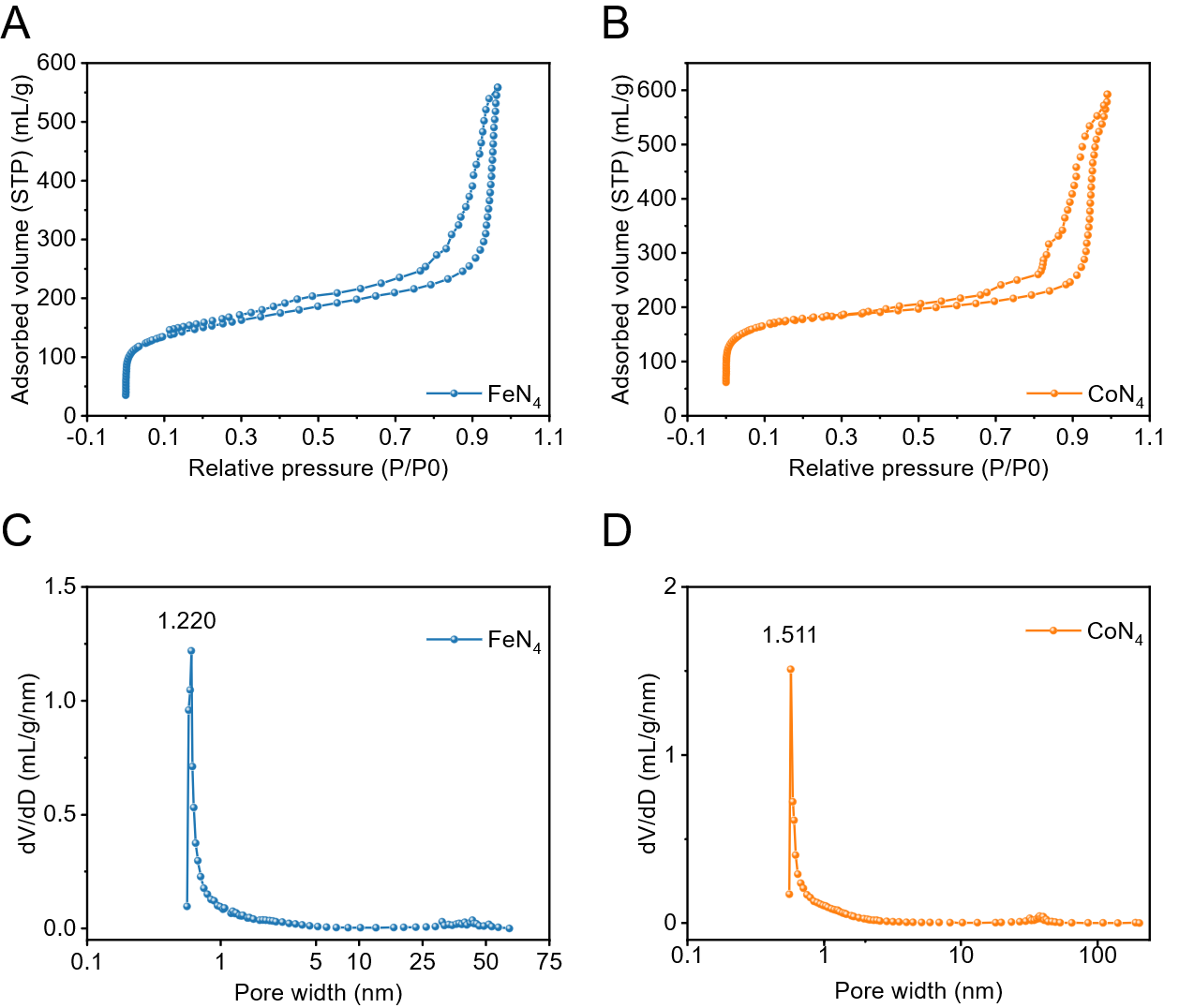


Figure S2 (A, B) N_2_ adsorption-desorption isotherms and (C, D) the corresponding pore size distribution curves derived from the NLDFT method for the FeN_4_ and CoN_4_ samples.


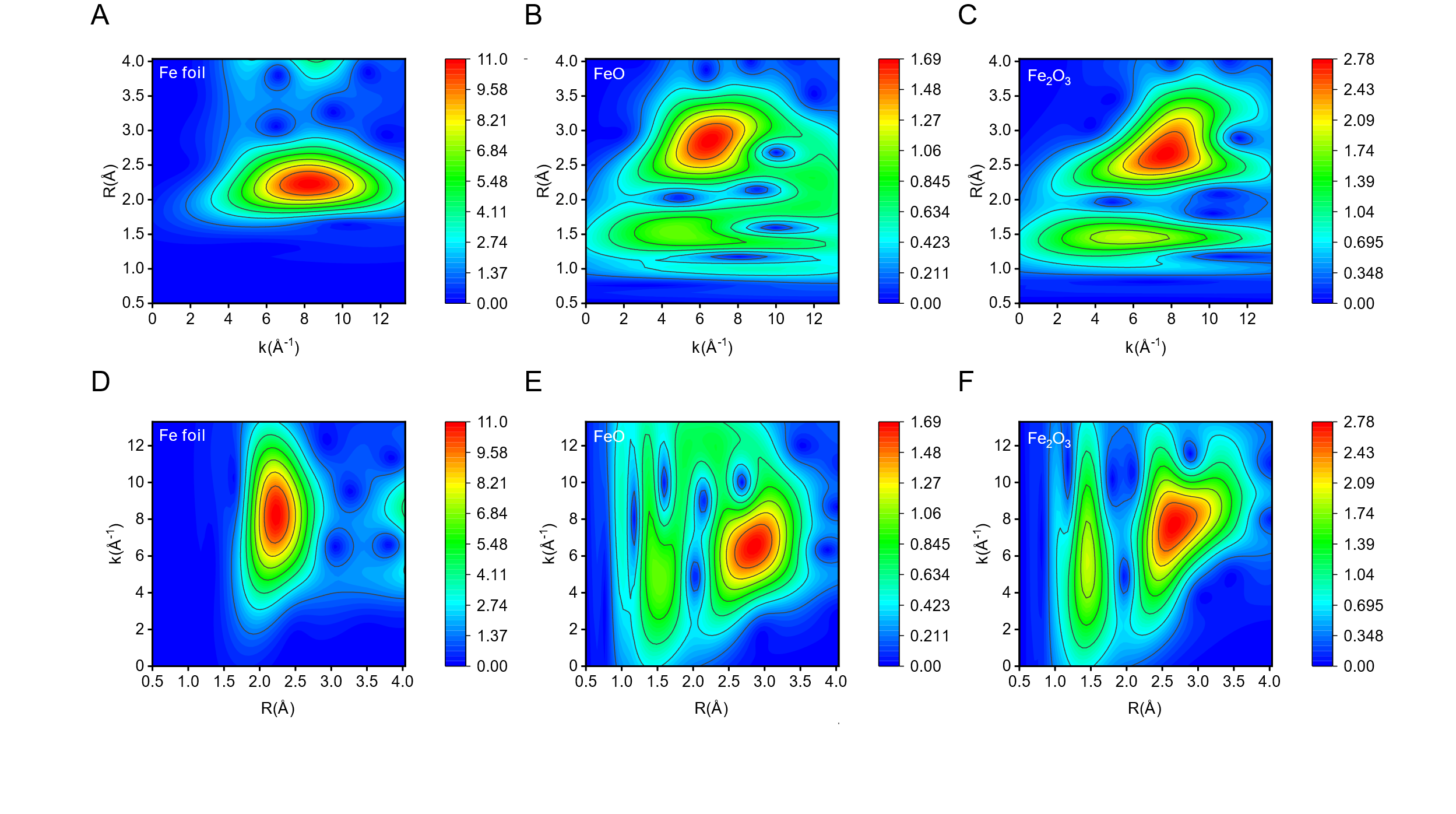


Figure S3 Wavelet Transform (WT) analysis of Fe K-edge EXAFS spectra. The WT contour plots are shown for the standard reference materials: (A, D) Fe foil, (B, E) FeO, and (C, F) Fe_2_O_3_. The transform resolves the EXAFS signals in both k-space (Å^-1^) and R-space (Å), helping distinguish different atomic scattering paths. The top and bottom rows represent the same data with transposed axes for enhanced clarity.


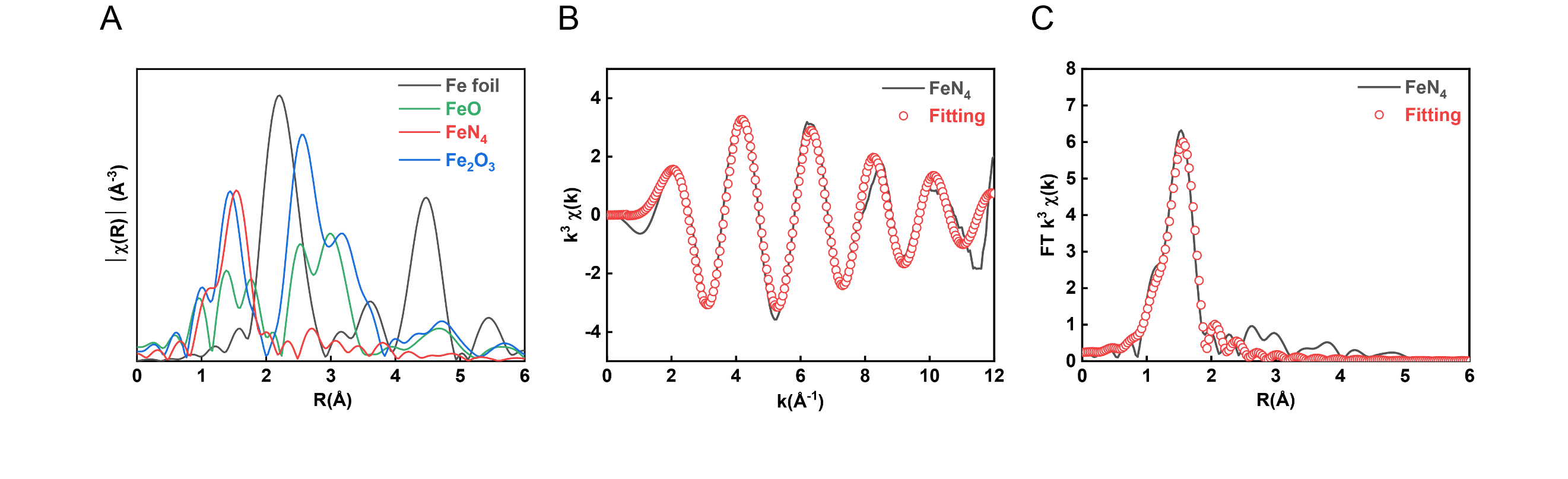


Figure S4 Local atomic structure analysis of the FeN_4_ catalyst using Fe K-edge EXAFS spectroscopy. (A) Fourier-transformed (FT) EXAFS spectra in R-space for FeN_4_ compared with reference standards of Fe foil, FeO, and Fe_2_O_3_. (B) k^3^-weighted EXAFS oscillation in k-space and (C) corresponding FT-EXAFS spectrum in R-space for FeN_4_. The solid black lines represent the experimental data, and the open red circles represent the best-fit results.


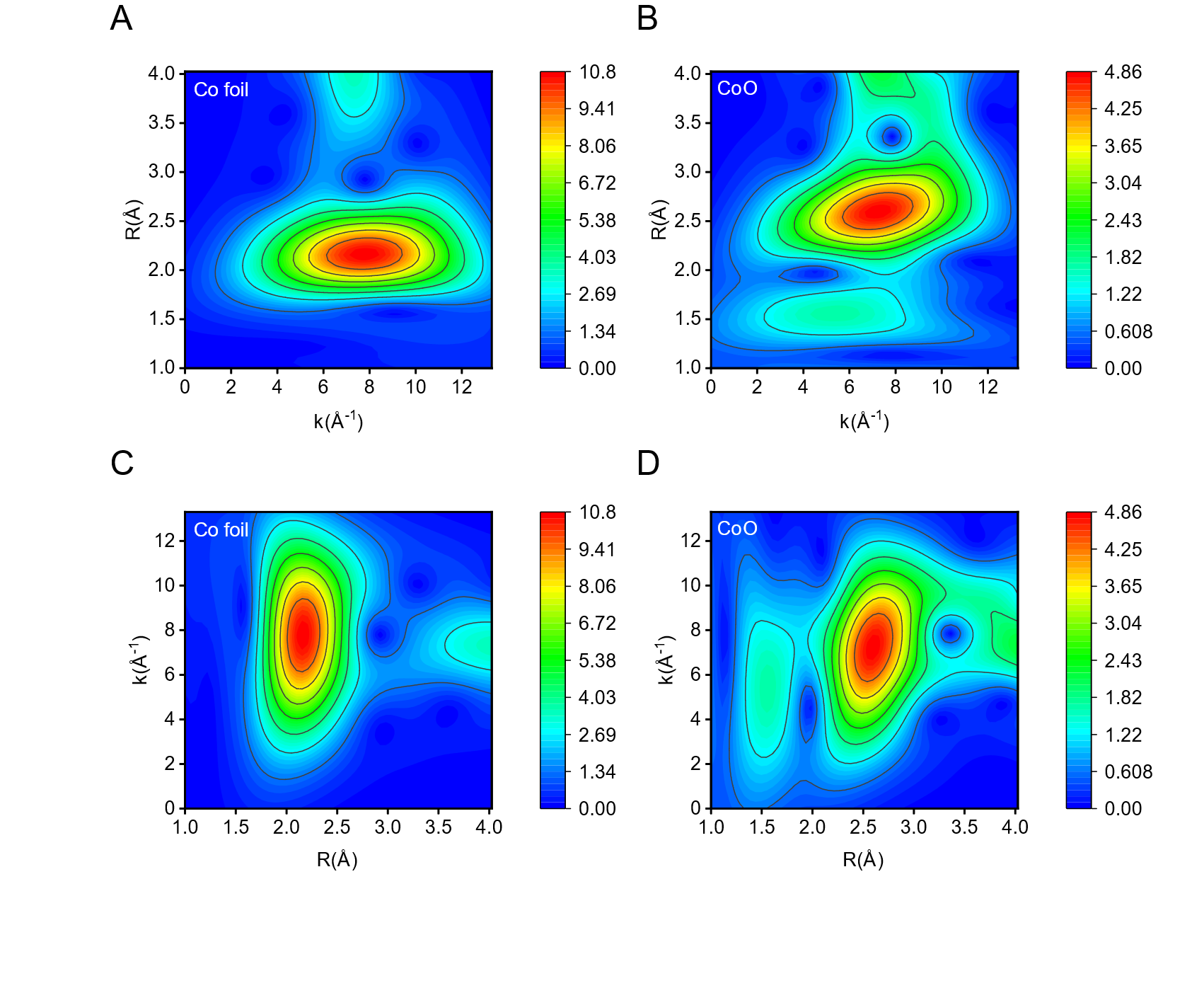


Figure S5 Wavelet Transform (WT) analysis of Co K-edge EXAFS spectra. The WT contour plots are shown for the standard reference materials: (A, C) Co foil and (B, D) CoO. The transform resolves the EXAFS signals in both k-space (Å^-1^) and R-space (Å), helping to distinguish different atomic scattering paths. The top and bottom rows represent the same data with transposed axes for clarity.


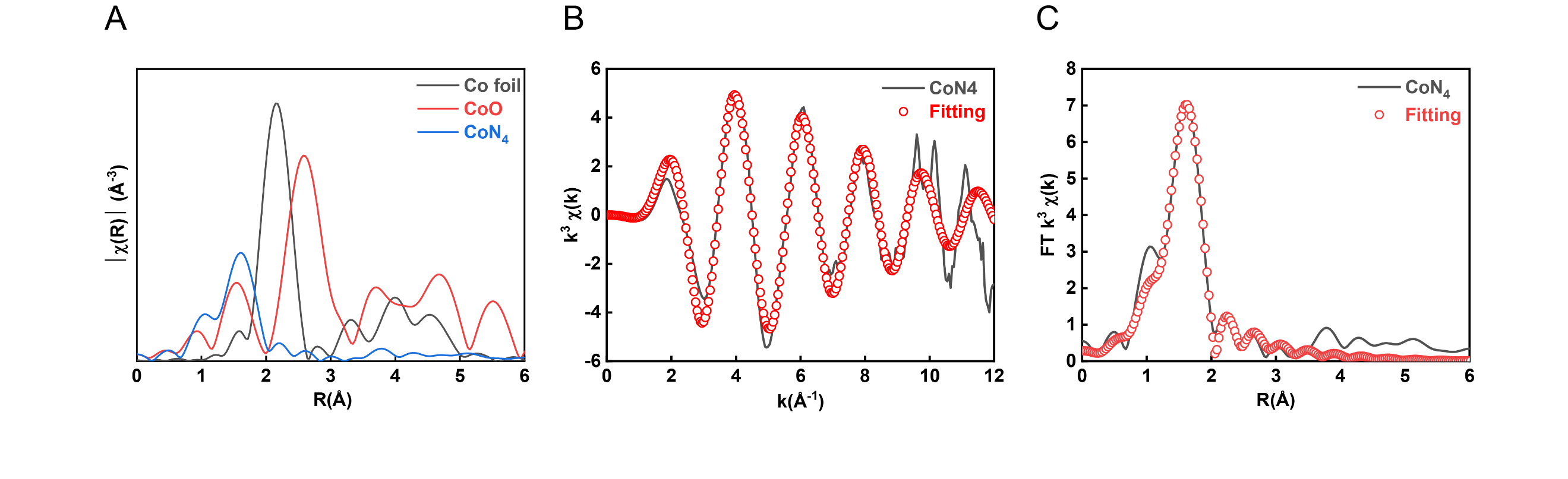


Figure S6 Local atomic structure analysis of the CoN_4_ catalyst using Co K-edge EXAFS spectroscopy. (A) Fourier-transformed (FT) EXAFS spectra in R-space for CoN_4_ compared with reference standards of Co foil and CoO. (B) k^3^-weighted EXAFS oscillation in k-space and (C) corresponding FT-EXAFS spectrum in R-space for CoN_4_. The solid black lines represent experimental data and the open red circles represent the best-fit results.

**
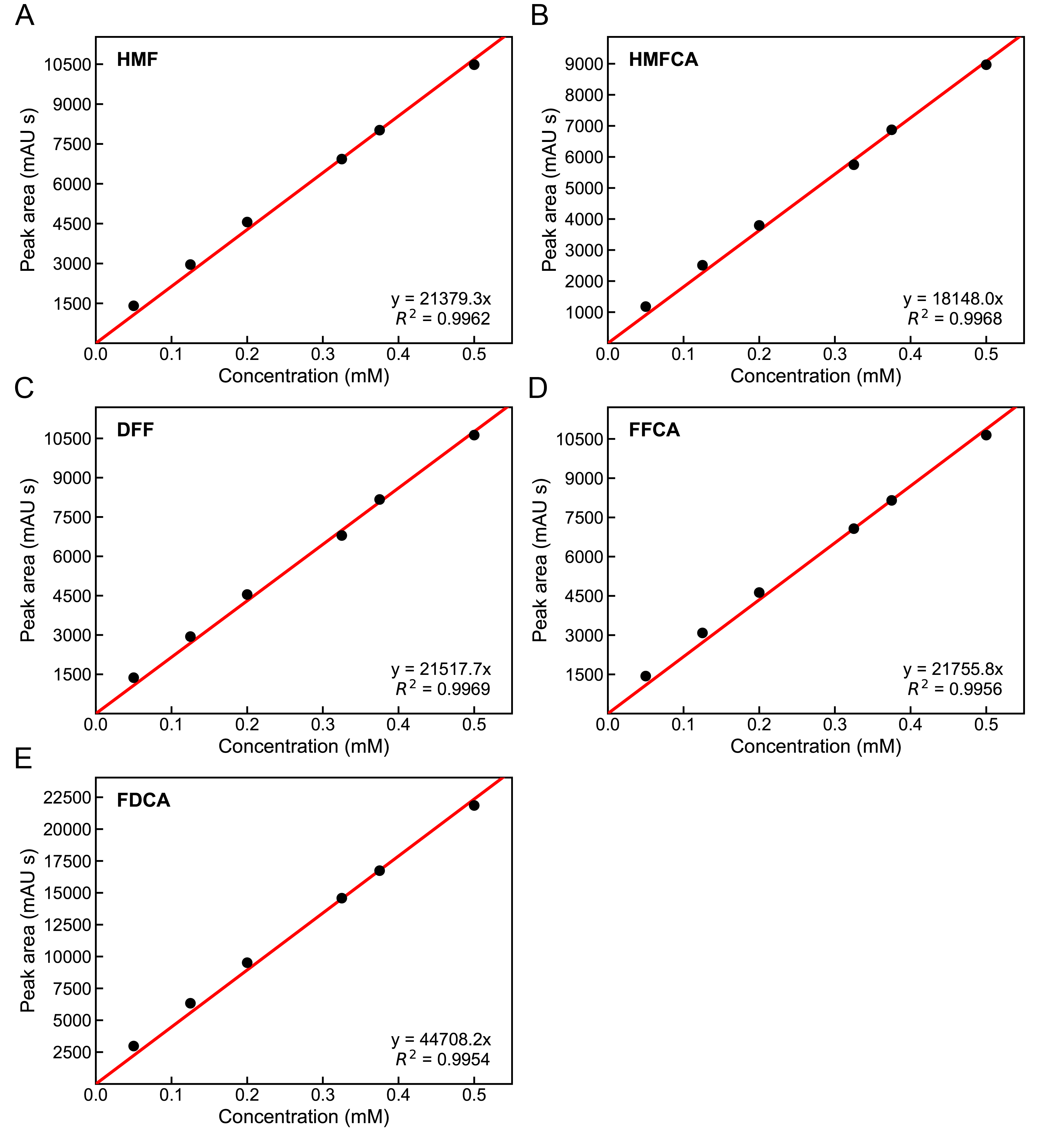
**

Figure S7 The HPLC external standard curves of pure (A) HMF; (B) HMFCA; (C) FFCA; (D) DFF and (E) FDCA.

**
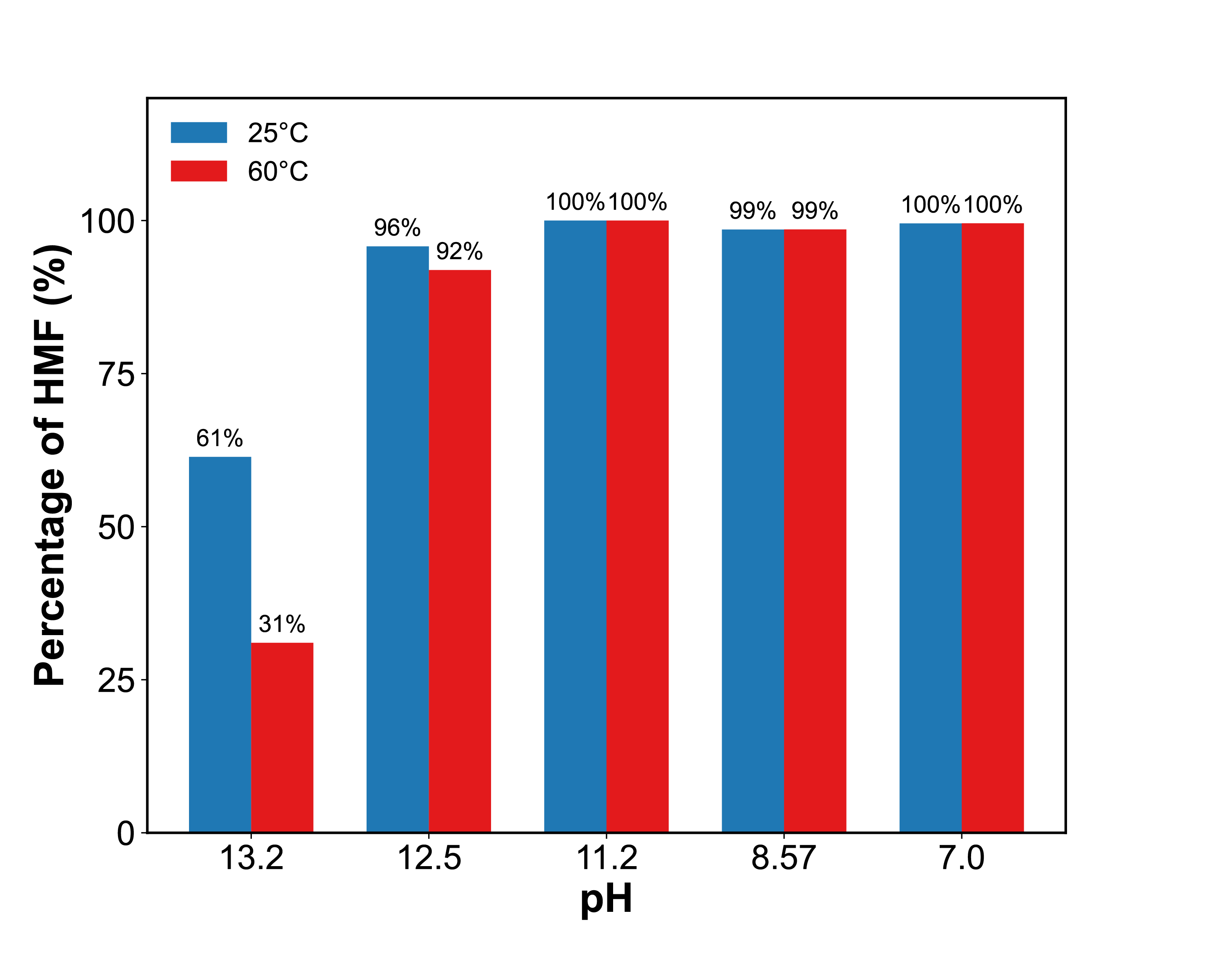
**

Figure S8 Effect of different reaction conditions on substrate conversion in the absence of a catalyst (blank experiment). Reaction conditions: 0.5 mmol HMF, 40 mg catalyst, and 100 mL H_2_O were added to the reactor with varying amounts of NaOH (50, 5, 0.5, 0.05, and 0 mmol). The reaction was conducted under a continuous O_2_ flow (50 mL/min) with a stirring rate of 1200 rpm.

**
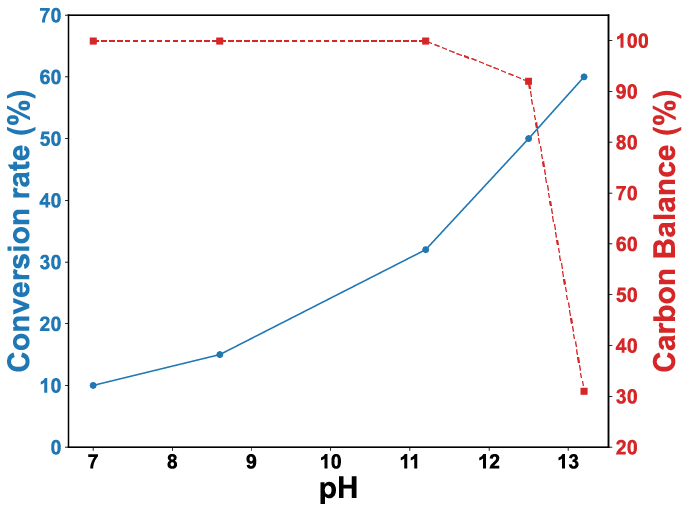
**

Figure S9 Optimization of the reaction conditions for the catalytic conversion of HMF. Effect of the initial pH on the HMF conversion rate (blue line) and carbon balance (red line) at 60 °C. General reaction conditions: 0.5 mmol HMF, 40 mg catalyst, 100 mL H_2_O, 4 h.


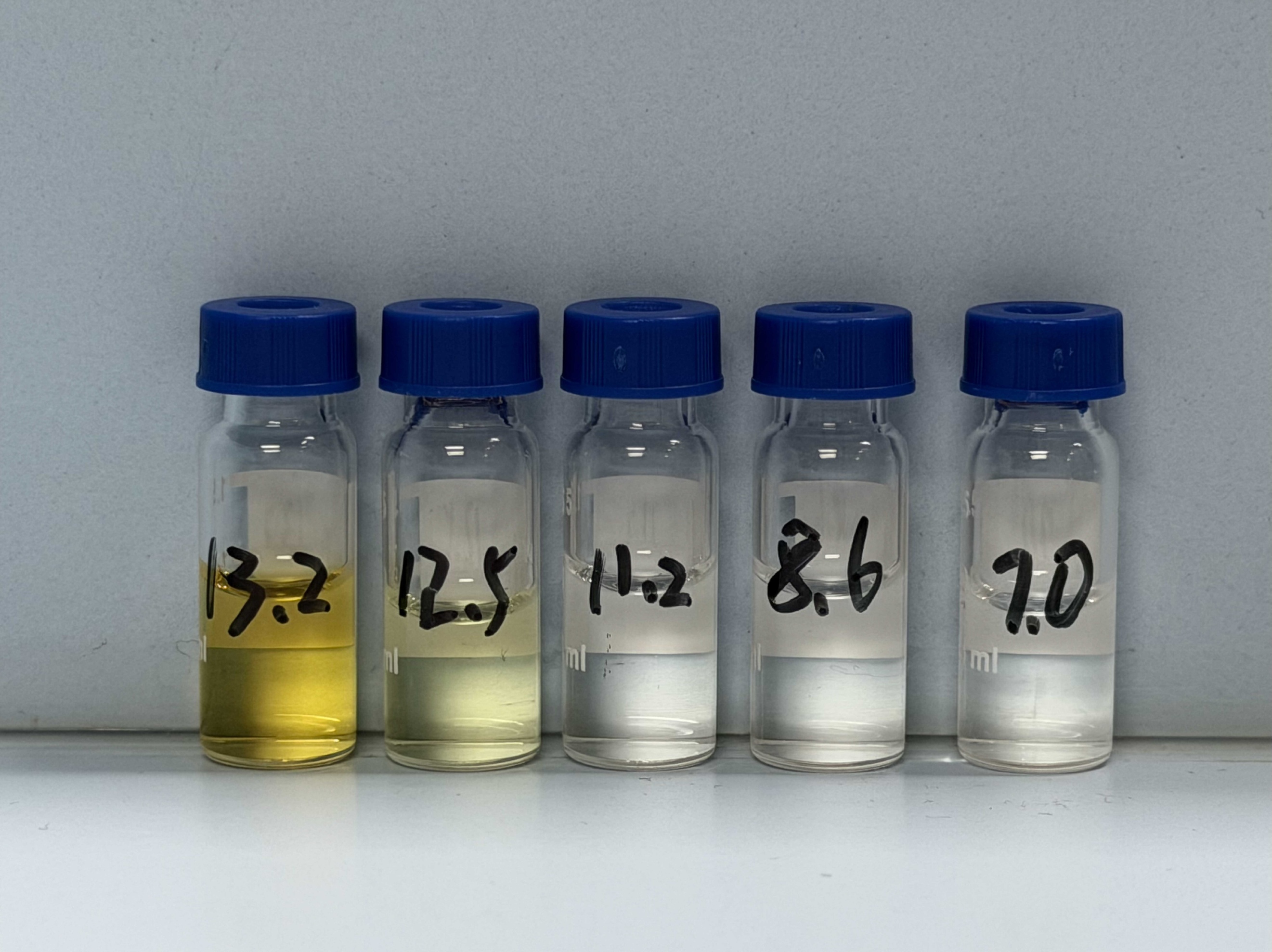


Figure S10 Solution color change under varying alkalinity conditions. Reaction conditions: 0.5 mmol HMF, 40 mg catalyst, and 100 mL H_2_O were added to the reactor with varying amounts of NaOH (50, 5, 0.5, 0.05, and 0 mmol). The reaction was conducted under a continuous O_2_ flow (50 mL/min) with a stirring rate of 1200 rpm.


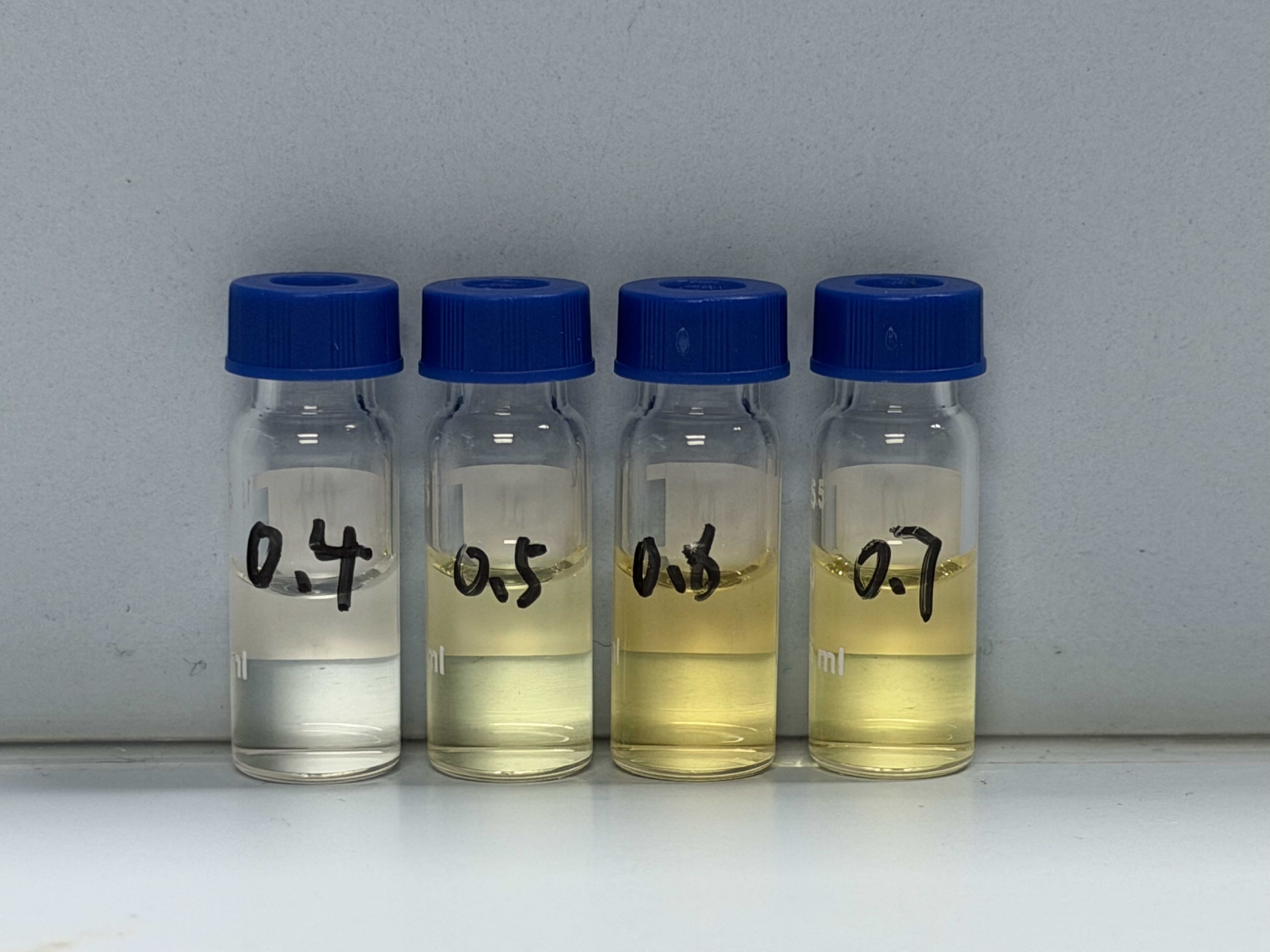


Figure S11 Solution color change under varying HMF concentration conditions. Reaction conditions: 5 mmol NaOH, 40 mg catalyst, and 100 mL H_2_O were used with varying amounts of HMF (0.4, 0.5, 0.6, and 0.7 mmol). The reaction was performed at 1200 rpm with a continuous O_2_ flow of 50 mL/min.


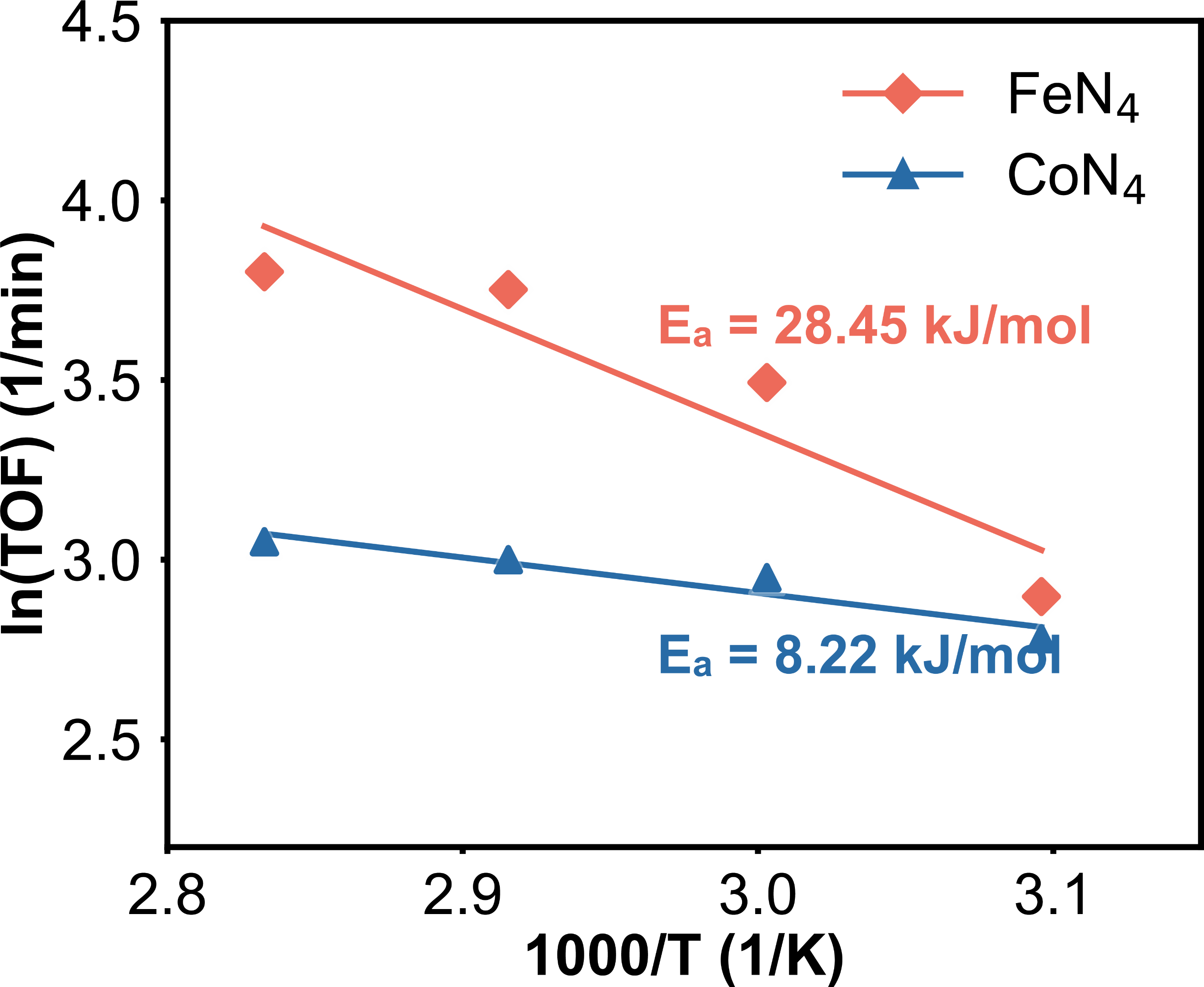


Figure S12 Arrhenius plots for the determination of apparent activation energies. Reaction conditions: 0.5 mmol HMF, 5 mmol NaOH, 40 mg catalyst, and 100 mL H_2_O were reacted at 40–70 °C (with 10 °C intervals) under a continuous O_2_ flow (50 mL/min) and 1200 rpm. The reaction time was carefully adjusted at each temperature to limit the HMF conversion within 30% for kinetic analysis.


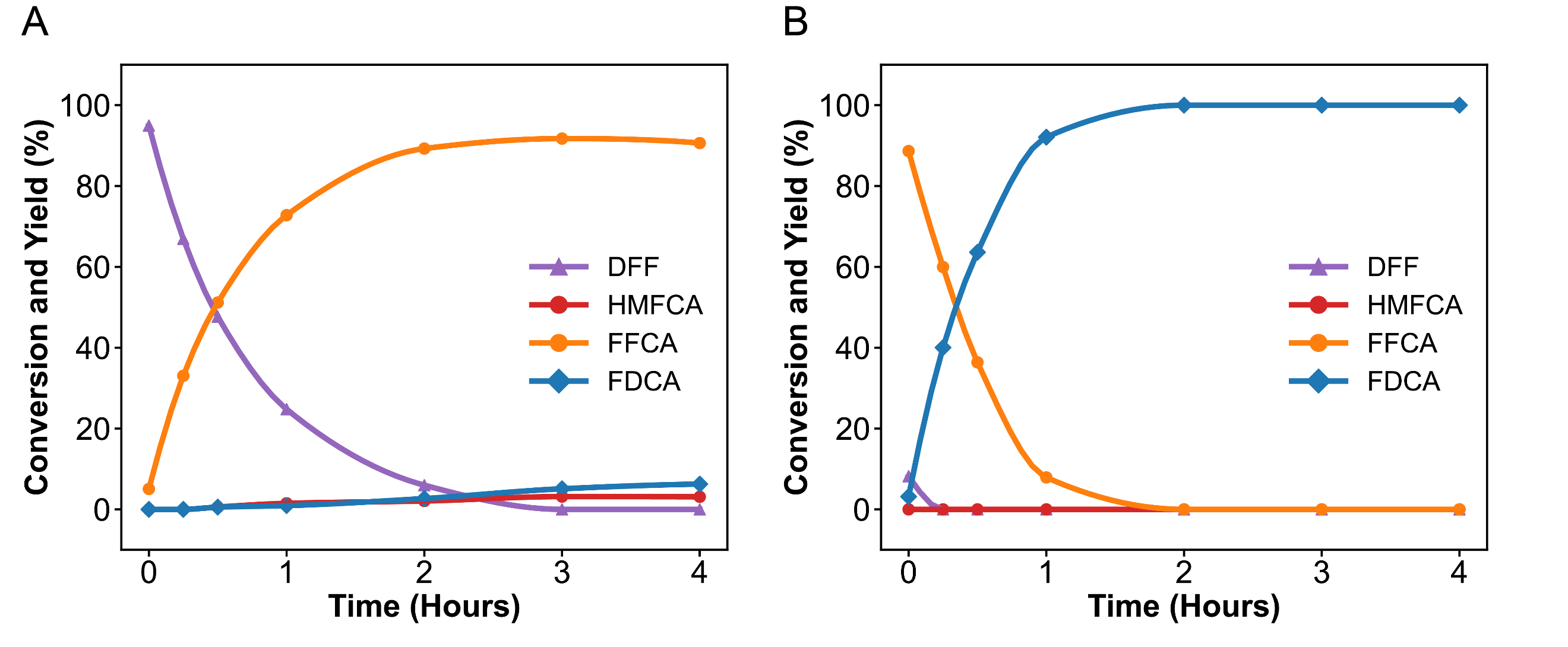


Figure S13 Catalytic performance of FeN_4_ and CoN_4_ for aerobic oxidation of DFF. The reaction profiles show the conversion of DFF and the yields of the key products over time. (A) Oxidation of DFF catalyzed by FeN_4_. (B) Oxidation of DFF catalyzed by CoN_4_. Reaction conditions: 0.5 mmol DFF, 60 °C, ambient pressure in an aqueous NaOH solution with a continuous O_2_ flow of 50 mL/min, stirring rate = 1200 rpm).


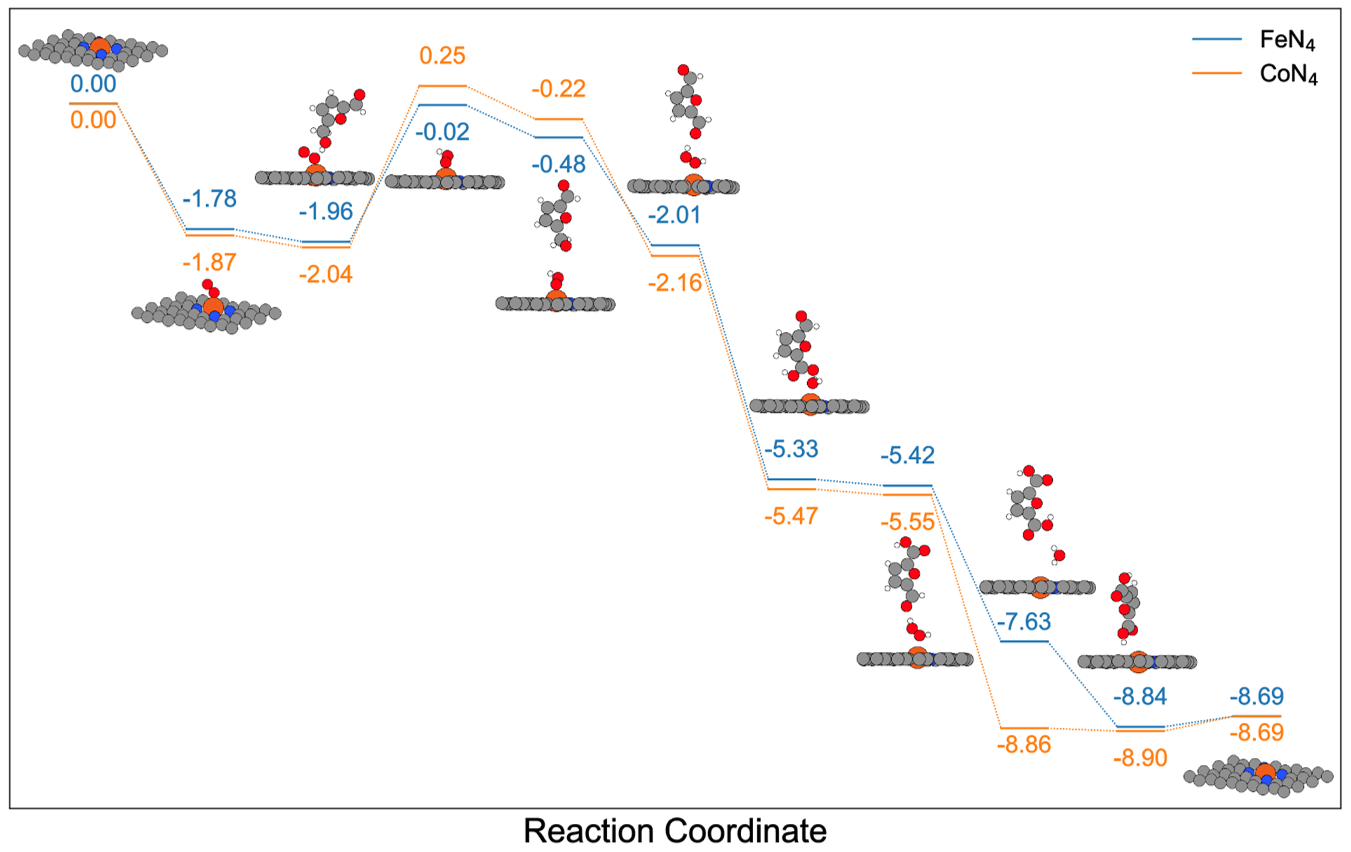


Figure S14 Free energy diagrams for the complete HMF-to-FDCA oxidation pathway on FeN_4_ (blue) and CoN_4_ (orange) active sites with explicit consideration of reactive oxygen and water species. The reaction proceeds through sequential O_2_ activation, *OOH-mediated aldehyde oxidation, and H_2_O_2_/H_2_O formation steps. Insets show the optimized adsorption configurations at each intermediate state. All energies are referenced to the initial state of the clean catalyst surface with gaseous O_2_ and HMF molecules.The elementary steps corresponding to the reaction coordinates are defined as follows (where * denotes an active site or an adsorbed state):

**Step 0:** * + O_2_ + HMF;

**Step 1:** * + O_2_* + HMF;

**Step 2:** O_2_* + HMF*;

**Step 3:** OOH* + RO;

**Step 4:** OOH* + RO*;

**Step 5:** H_2_O_2_* + DFF*;

**Step 6:** H_2_O* + FFCA*;

**Step 7:** H_2_O_2_* + FFCA*;

**Step 8:** FDCA* + H_2_O *;

**Step 9:** FDCA* + H_2_O;

**Step 10:** * + FDCA + H_2_O.


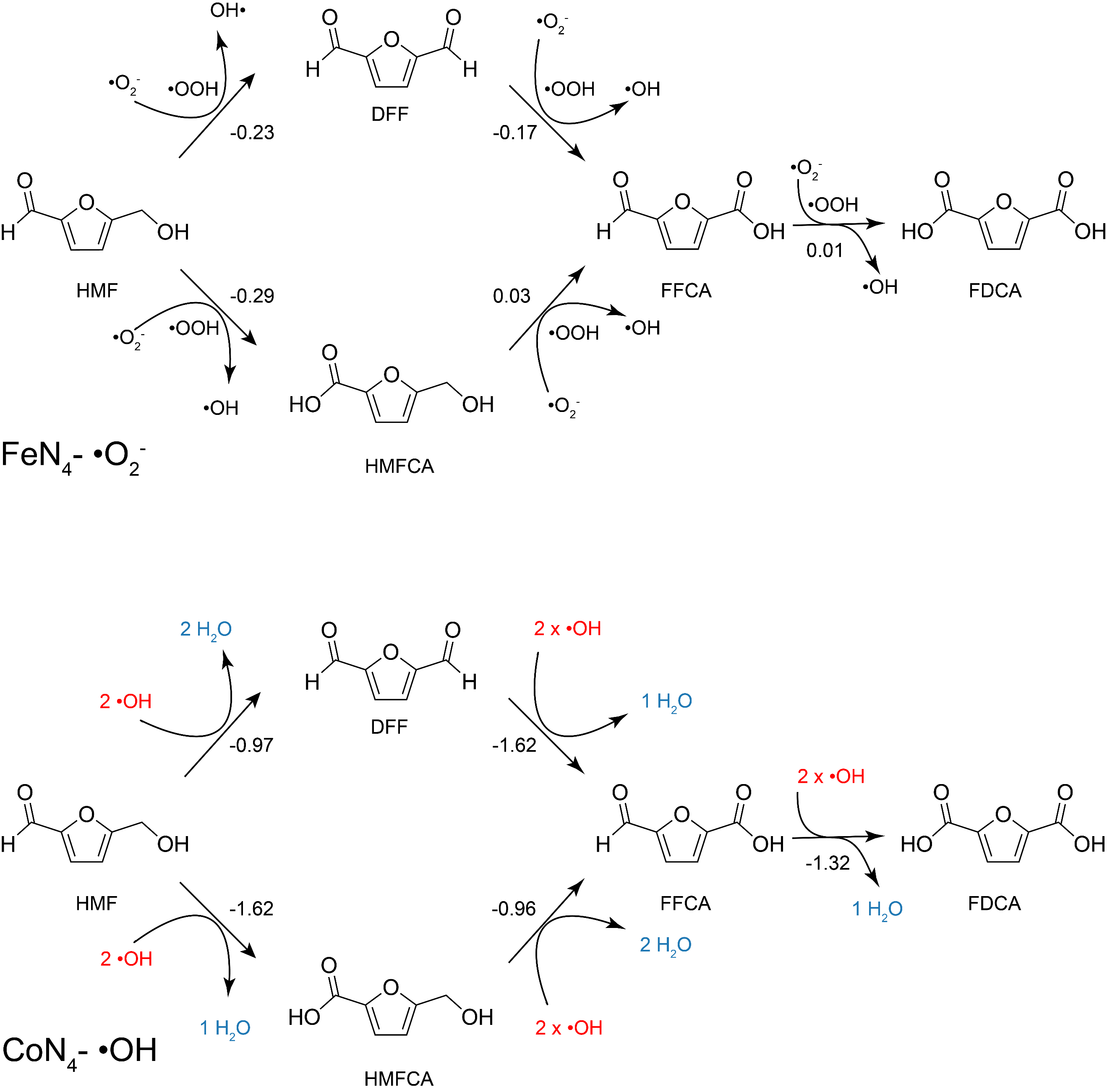


Figure S15 Calculated reaction energy profiles for the HMF oxidation pathways on the FeN_4_ site with O_2_⁻ and the CoN₄ site with •OH.


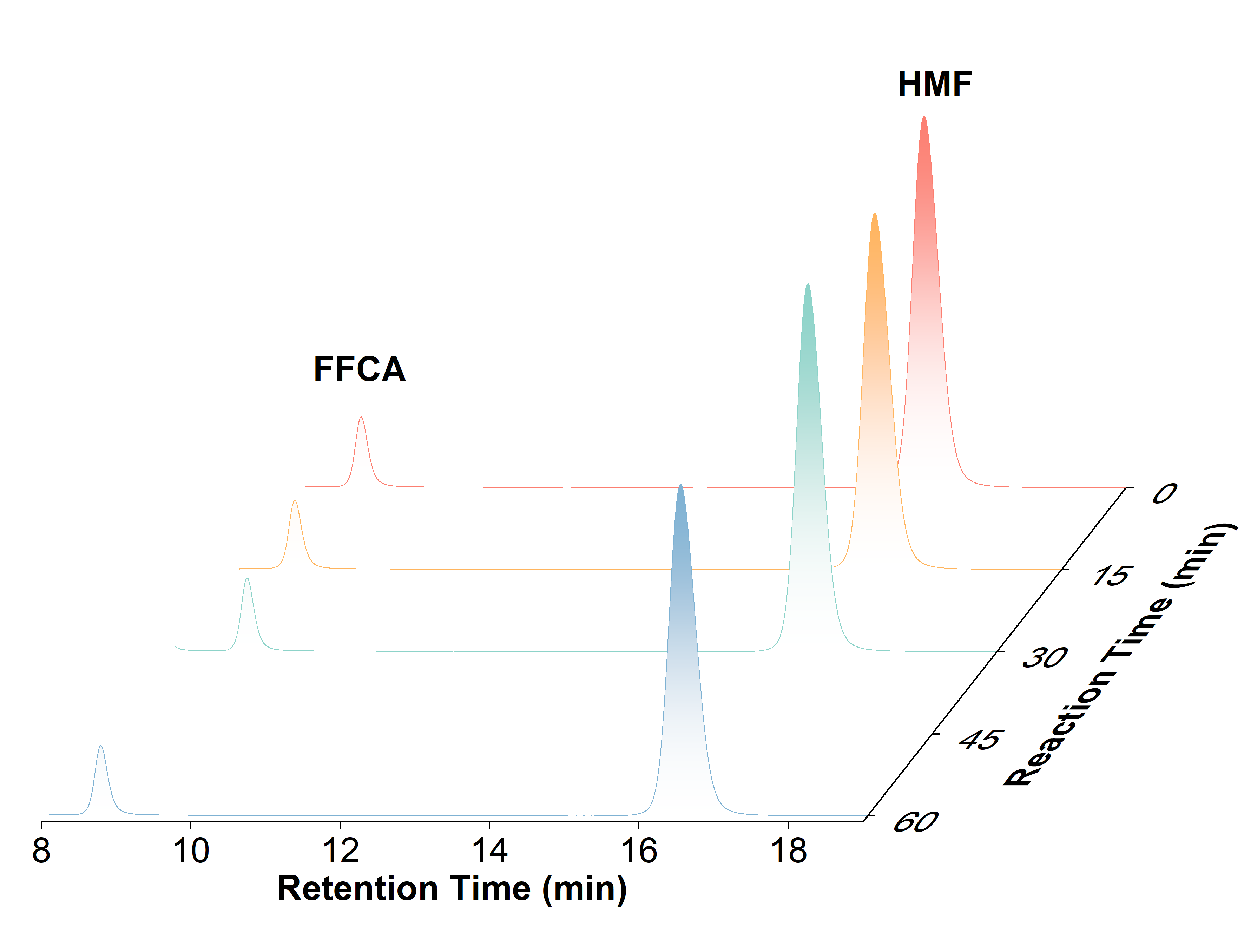


Figure S16 Time-resolved HPLC chromatograms illustrating the product evolution during HMF oxidation. The chromatograms have been background-subtracted to eliminate dissolved ion signals. Reaction conditions: A 10 mL mixture consisting of 5 mL of the leaching solution and 5 mL of an aqueous solution containing 0.25 mmol HMF and 0.25 mmol NaOH, 60 °C, ambient pressure, with a continuous O_2_ flow of 50 mL/min.

**Supplementary table**

Table. S1 Elemental Composition of FeN_4_ and CoN_4_ Determined by ICP Analysis

| **Sample** | **Metal Content (wt.%)** |
| --- | --- |
| FeN_4_ | 0.99% |
| CoN_4_ | 2.03% |

Table. S2 Textural properties of the CoN_4_ and FeN_4_ samples

| **parameter** | **CoN_4_** | **FeN_4_** |
| --- | --- | --- |
| BET surface area / m^2^ g^-1^ | 573 | 502 |
| Langmuir surface area / m^2^ g^-1^ | 834 | 751 |
| T‑plot micropore area / m^2^ g^-1^ | 458 (80 % of BET) | 208 (41 %) |
| T‑plot external area / m^2^ g^-1^ | 115 | 293 |
| total pore volume / cm^3^ g^-1^ | 0.909 | 0.864 |
| micropore volume / cm^3^ g^-1^ | 0.230 (25 %) | 0.104 (12 %) |
| BJH desorption mean pore dia. / nm | 11.8 | 8.8 |
| 4V/A (BET) average pore dia. / nm | 6.35 | 6.89 |

Table. S3 Summary of EXAFS fitting parameters for the FeN_4_ and CoN_4_ samples

| Sample | Scattering Path | CN | σ^2^ (Å^2^) | ΔE_0_ (eV) |
| --- | --- | --- | --- | --- |
| FeN_4_ | Fe-N | 3.9 ± 0.4 | 0.0065 ± 0.0020 | 1.4 ± 1.1 |
| CoN_4_ | Co-N | 4.1 ± 0.6 | 0.0075 ± 0.0017 | 0.3 ± 1.2 |

Table. S4 Comparison of TOF values and reaction conditions for HMF oxidation

| Sample | TOF (h^-1^) |
| --- | --- |
| FeN_4_ | 18.12 |
| CoN_4_ | 18.42 |

Table. S5 ICP-OES Analysis of Post-Reaction Filtrates (Metal Leaching Data)

| Sample | **Metal ion concentration (μg/L)** |
| --- | --- |
| FeN_4__Cycle1 | 244.95 |
| FeN_4__Cycle2 | 232.56 |
| FeN_4__Cycle3 | 125.84 |
| CoN_4__Cycle1 | 8.74 |
| CoN_4__Cycle2 | 12.88 |
| CoN_4__Cycle3 | 9.09 |

**Table.** **S6 Overview of Reported Catalytic Systems for the Oxidative Conversion of HMF to FDCA**

| Catalyst | Support | Temperature (°C) | O_2_​ Pressure (MPa) | Solvent | Base (molar ratio to HMF) | Time (h) | HMF Conversion (%) | FDCA Yield/Selectivity (%) | Source |
| --- | --- | --- | --- | --- | --- | --- | --- | --- | --- |
| Pt/C | Carbon | 120 | 1 | Water | None | 2.5 | 100 | 89.7 (Yield) | [1] |
| Pt/NC-800 | N-doped carbon | 110 | 0.5 | Water | NaHCO_3_ | 24 | 100 | 83.9 (Yield) | [2] |
| Au-Pd/ZrO_2_ | ZrO_2_ | 80 | 0.5 | Water | NaHCO_3_ | 24 | 100 | 77.9 (Yield) | [3] |
| Au/MnO_2_ (5.6 wt%) | MnO_2_ | 100 | 0.1 | Water | None | 12 | 99 | 93.2 (Yield) | [4] |
| Au-Ni/MgAlOₓ | MgAlOₓ | 90 | 0.5 | Water | None | 2 | 100 | 76.6 (Yield) | [5] |
| Ru/C | Carbon | 110 | 1 | Water | Mg(OH)_2_ | 8 | 100 | 97.3 (Yield) | [6] |
| Ru/Cr_2_-Fe₁-O (4 wt%) | Cr_2_-Fe₁-O | 100 | 1 | Water | KHCO_3_ | 16 | 100 | 99.9 (Yield) | [7] |
| Ag/CeO_2_-DTPA-400 | CeO_2_ | Room Temp | 0.1 (O_2_) | Water | None | 6 | >99 | 98 (Selectivity) | [8] |
| MnFe_2_O_4_ | - | 100 | - | TBHP | - | 6 | - | 85 (Yield) | [9] |
| MnOₓ-VC | Carbon | 120 | 1 (O_2_​) | O_2_​ | Water | 4 | 100 | 99 (Yield) | [10] |
| V-MoO_3_ | - | - | - | TBHP | TBA | 10 | 99.5 | 87.4 (Selectivity) | [11] |
| CoOₓ-CeO_2_ | - | 130 | 0.6 (O_2_​) | O_2_​ | Water | 4 | 100 | 86.3 (Yield) | [12] |
| NiO/ZSM-5 | ZSM-5 | 130 | Ambient | H_2_O_2_ | Water | 5 | 100 | 79 (Yield) | [13] |

**Reference**

[1] G.Y. Ryu, D. Park, Y. Jo, C.C. Truong, D.K. Mishra, Y.-W. Suh, Ether-type co-solvents of excellence for aqueous base-free Pt-catalyzed oxidation of concentrated 5-(hydroxymethyl)furfural to 2,5-furandicarboxylic acid, Biomass Bioenergy 180 (2024) 107022. https://doi.org/10.1016/j.biombioe.2023.107022.

[2] C. Yang, X. Li, Z. Zhang, B. Lv, J. Li, Z. Liu, W. Zhu, F. Tao, G. Lv, Y. Yang, Utilization of biomass waste: Facile synthesis high nitrogen-doped porous carbon from pomelo peel and used as catalyst support for aerobic oxidation of 5-hydroxymethylfurfural, Fuel 278 (2020) 118361. https://doi.org/10.1016/j.fuel.2020.118361.

[3] T.S. Kharlamova, K.L. Timofeev, D.P. Morilov, M.A. Salaev, A.I. Stadnichenko, O.A. Stonkus, O.V. Vodyankina, Design strategy for effective supported Au–Pd catalysts for selective oxidation of 5-hydroxymethylfurfural under mild conditions, React. Chem. Eng. 9 (2024) 2691–2709. https://doi.org/10.1039/D4RE00355A.

[4] D. Zeng, W. Wang, B. Cui, B. Jiang, C. Zhang, L. Zhang, W. Wang, Base-free selective oxidation of 5-hydroxymethylfurfural to 2,5-furandicarboxylic acid over Au/MnO2 catalyst, Fuel 381 (2025) 133238. https://doi.org/10.1016/j.fuel.2024.133238.

[5] J. Su, Z. Liu, Y. Tan, Y. Xiao, N. Zhan, Y. Ding, Au-Based Bimetallic Catalysts for Aerobic Oxidation of 5-Hydroxymethylfurfural to 2,5-Furandicarboxylic Acid under Base-Free Reaction Conditions, Molecules 29 (2024) 2724. https://doi.org/10.3390/molecules29122724.

[6] L. Zheng, J. Zhao, Z. Du, B. Zong, H. Liu, Efficient aerobic oxidation of 5-hydroxymethylfurfural to 2,5-furandicarboxylic acid on Ru/C catalysts, Sci. China Chem. 60 (2017) 950–957. https://doi.org/10.1007/s11426-016-0489-3.

[7] S. Zhang, Y. Yang, G. Chu, S. Wang, C. Wang, Y. Zhang, L. Zhang, J. Mei, Surface chemistry regulation on particle-support interaction of ruthenium and Cr-Fe oxides for selective oxidation of 5-hydroxymethylfurfural, Chem. Eng. J. 474 (2023) 145670. https://doi.org/10.1016/j.cej.2023.145670.

[8] S. Zhao, K. Wang, S. Mao, B. Yang, Y. Zhao, Toward Maximizing the Ag–CeO_2_ Interface and Rich Oxygen Vacancies for Efficient Photocatalytic Oxidation of 5-Hydroxymethylfurfural, ACS Sustain. Chem. Eng. 13 (2025) 1081–1094. https://doi.org/10.1021/acssuschemeng.4c09208.

[9] A.B. Gawade, A.V. Nakhate, G.D. Yadav, Selective synthesis of 2, 5-furandicarboxylic acid by oxidation of 5-hydroxymethylfurfural over MnFe 2 O 4 catalyst, Catal. Today 309 (2018) 119–125. https://doi.org/10.1016/j.cattod.2017.08.061.

[10] J. Wu, W. Xie, Y. Zhang, X. Ke, T. Li, H. Fang, Y. Sun, X. Zeng, L. Lin, X. Tang, Oxygen-vacancy-rich MnO supported RuO for efficient base-free oxidation of 5-hydroxymethylfurfural and 5-methoxymethylfurfural to 2,5-furandicarboxylic acid, J. Energy Chem. 95 (2024) 670–683. https://doi.org/10.1016/j.jechem.2024.04.019.

[11] L. Yang, J. Liu, F. Cheng, S. Zhou, Q. Xu, D. Yin, X. Liu, V-doped MoO3 nanorods for highly selective oxidation of 5-hydroxymethylfurfural to bio-monomer 2, 5-furandicarboxylic acid, Renew. Energy 226 (2024) 120409. https://doi.org/10.1016/j.renene.2024.120409.

[12] M. Jin, L. Yu, H. Chen, X. Ma, K. Cui, Z. Wen, Z. Ma, Y. Sang, M. Chen, Y. Li, Base-free selective conversion of 5-hydroxymethylfurfural to 2,5-furandicarboxylic acid over a CoOx-CeO2 catalyst, Catal. Today 367 (2021) 2–8. https://doi.org/10.1016/j.cattod.2020.10.038.

[13] I. Herlina, Y.K. Krisnandi, M. Ridwan, Oxidation of 5-hydroxymethylfurfural into 2,5-furandicarboxylic acid over CuO and NiO modified natural sourced hierarchical ZSM-5, South Afr. J. Chem. Eng. 47 (2024) 75–82. https://doi.org/10.1016/j.sajce.2023.10.011.
